# Supplementary material for: Tissue Mechanics Regulate Mitotic Nuclear Dynamics during Epithelial Development
Source: Curr Biol. 2020 Jul 6;30(13):2419–2432.e4. doi: 10.1016/j.cub.2020.04.041 (PMC7342018; doi:10.1016/j.cub.2020.04.041)
Supplement: Document S1. Figures S1–S7 [file mmc1.pdf]

Current Biology, Volume *30*

# Supplemental Information

# Tissue Mechanics Regulate Mitotic Nuclear Dynamics during Epithelial Development

**Natalie J. Kirkland, Alice C. Yuen, Melda Tozluoglu, Nancy Hui, Ewa K. Paluch, and Yanlan Mao**

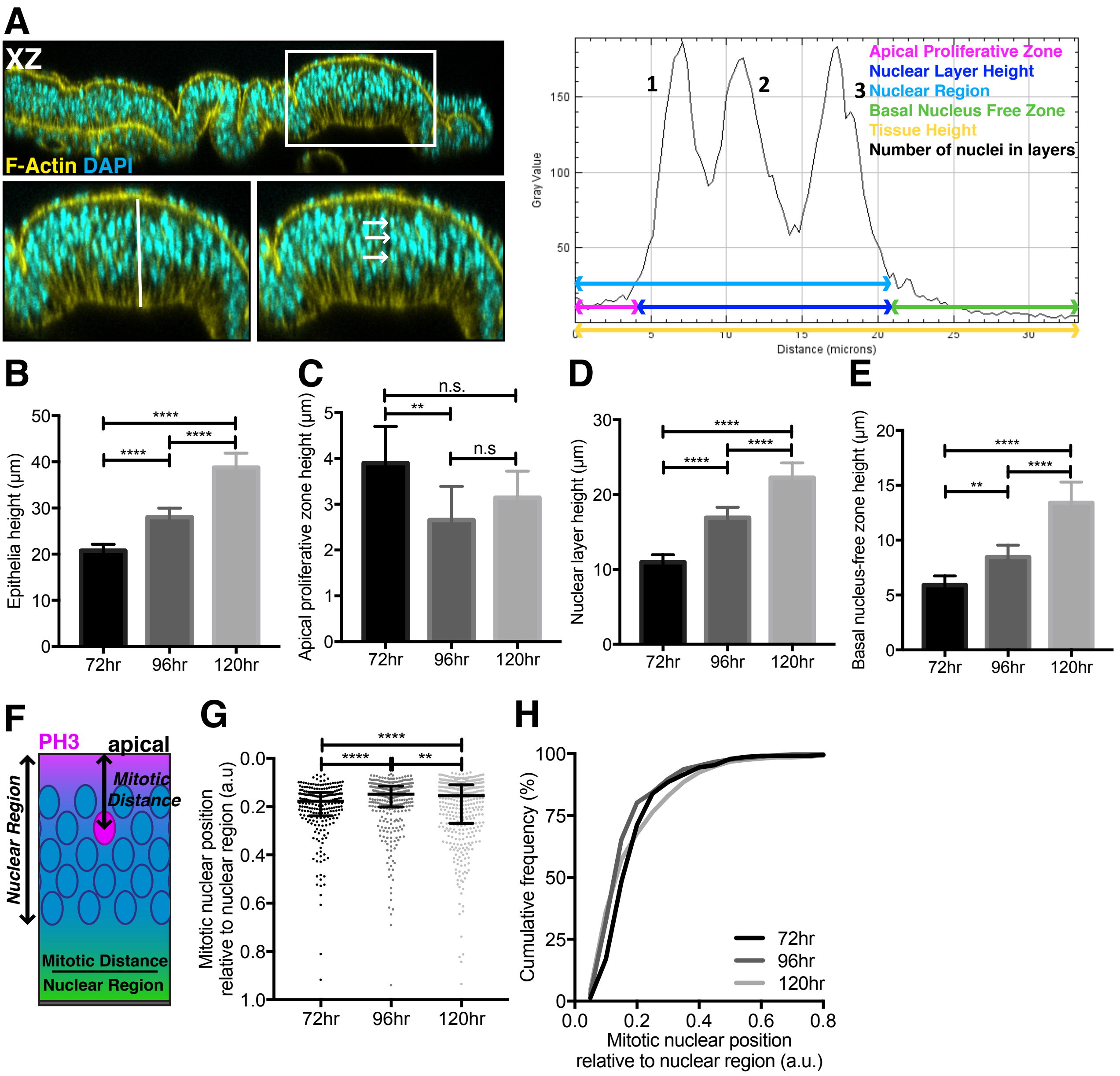


**Figure S1. Changes in the wing disc architecture during development are associated with distinct patterns of mitotic nuclear positioning. Related to Figure 1.**

(A) Left panel, lateral cross section of wing disc fixed and stained with DAPI (cyan) and phalloidin (yellow). White box highlights inset pouch region shown below. White line in lower left inset highlights the apico-basal cross-section used to generate the intensity plot displayed in the right panel. Lower right inset: white arrows mark the nuclei shown as peaks in the right panel, used for counting nuclei number in nuclear layer. Right panel: labelled intensity plot illustrating quantification for Figure 1B-C and S1B-E. (B-E) Epithelial and sub-region height, also presented in Figure 1C. (F) Schematic showing mitotic nuclear position relative to the nuclear region as in S1F-H. (G-H) Normalised PH3+ distance from Figure 1G-H presented as dot plot (G) and cumulative frequency distribution (H). Average nuclear region height calculated per disc. (B-E, G-H) n = 8 (72hr), 8 (96hr) and 5 (120hr) wing. Statistical significance: B-E, one-way ANOVA; G, Kolmogorov-Smirnov comparison of cumulative distribution. n.s.>0.05, **p<0.01, ****p<0.0001. Error bars: B-E, mean ± SD; G, median ± interquartile range.

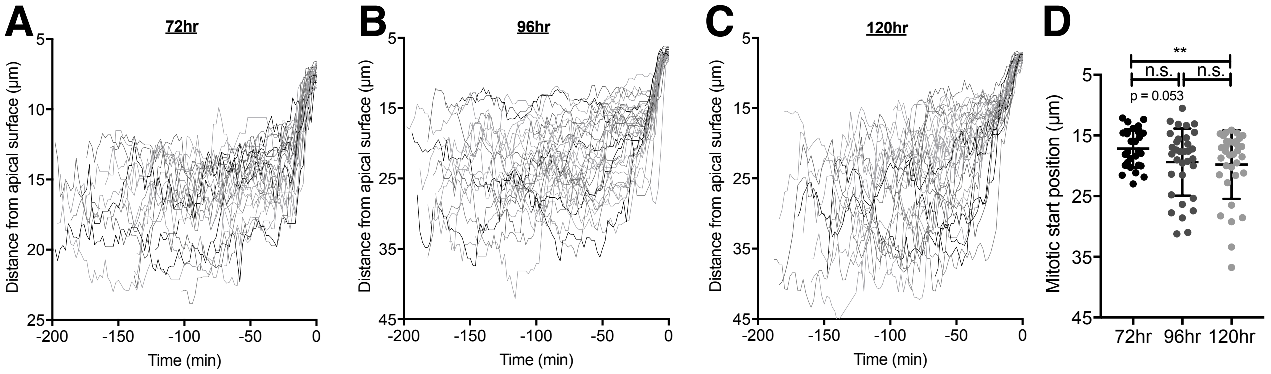


**Figure S2. Mitotic nuclear dynamics depend on tissue architecture. Related to Figure 2.**
(A-C) Individual nuclei tracks from wing disc pouch region at 72, 96 and 120 hours AEL, expressing Sqh-GFP and His-RFP, obtained by measuring distances illustrated in Figure 2A-C. 0 minutes is defined by metaphase nucleus prior to anaphase separation. (D) The position at the onset of apical nuclear movement, used to calculate mitotic nuclear velocity in Figure 2D. Statistical significance: one-way ANOVA. Error bars: mean ± SD. (A-D) n = 28, 33 and 34 nuclear trajectories from 3 (72hr), 3 (96hr) and 4 (120hr) wing discs respectively. n.s. $p>0.05$, **$p<0.01$.

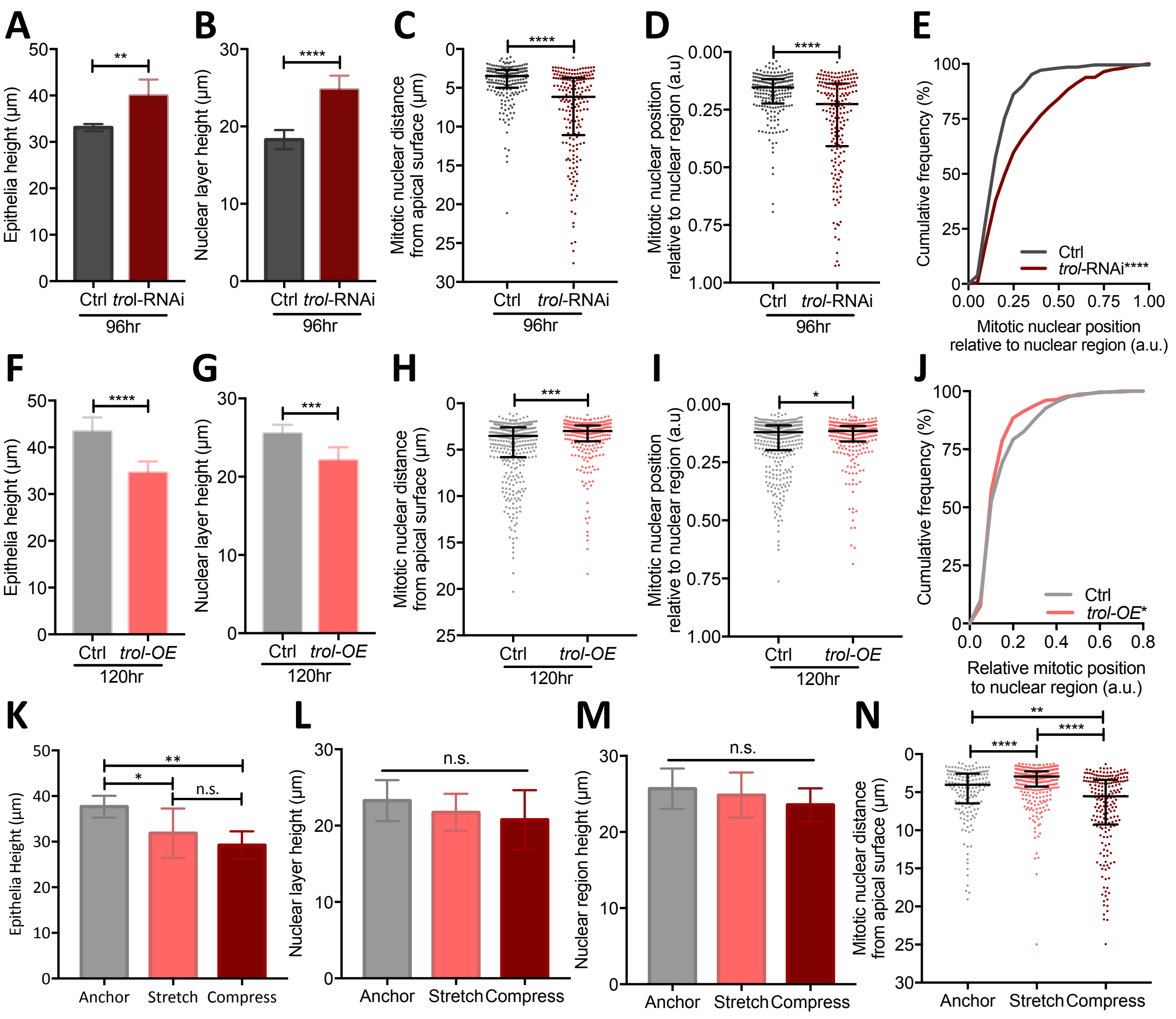


**Figure S3. Perturbing cell density influences mitotic nuclear positioning. Related to Figure 3.**

(A, B) Quantification of apico-basal epithelial morphology for control (*actin*Gal4) and *UAS-trol-RNAi* (*trol-RNAi*) wing discs, corresponding to Figure 3B. (C) Scatter plot of mitotic nuclear distance from the apical surface for control and *trol-RNAi* wing discs, corresponding to Figure 3D. (D, E) Mitotic nuclear position relative to the nuclear region for control and *trol-RNAi* expressing wing discs presented as scatter plot (D) and cumulative frequency distribution (E). (F, G) Quantification of apico-basal epithelial morphology for control and *UAS-trol (trol-OE)* wing discs, corresponding to Figure 3F. (H) Scatter plot of mitotic nuclear distance from the apical surface for control and *trol-OE* wing discs, corresponding to Figure 3H. (I, J) Mitotic nuclear position relative to the nuclear region for control and *trol-OE* expressing wing discs presented as scatter plot (I) and cumulative frequency distribution (J). (K-M) Quantification of apico-basal epithelial morphology in the wing disc pouch region upon mechanical perturbation as illustrated in Figure 3E. (N) Scatter plot of mitotic nuclear distance from the apical surface upon mechanical perturbation, corresponding to Figure 3I. (A-E) n = 6 (control) and 7 (*trol-RNAi*) wing discs. (F-J) n = 8 (control) and 11 (*trol-OE*) wing disc discs. (K-N) n = 3 (anchored), 8 (stretched) and 6 (compressed) wing discs. Statistical significance: A, B, F, G, unpaired t-test; C-E, H-J, N, Kolmogorov-Smirnov comparison of cumulative distribution; K-M, one-way ANOVA. *$p<0.05$, **$p<0.01$, ***$p<0.001$, ****$p<0.0001$. Error bars: A, B, F, G, K-M, mean ± SD; C, D, H, I, N, median ± interquartile range.

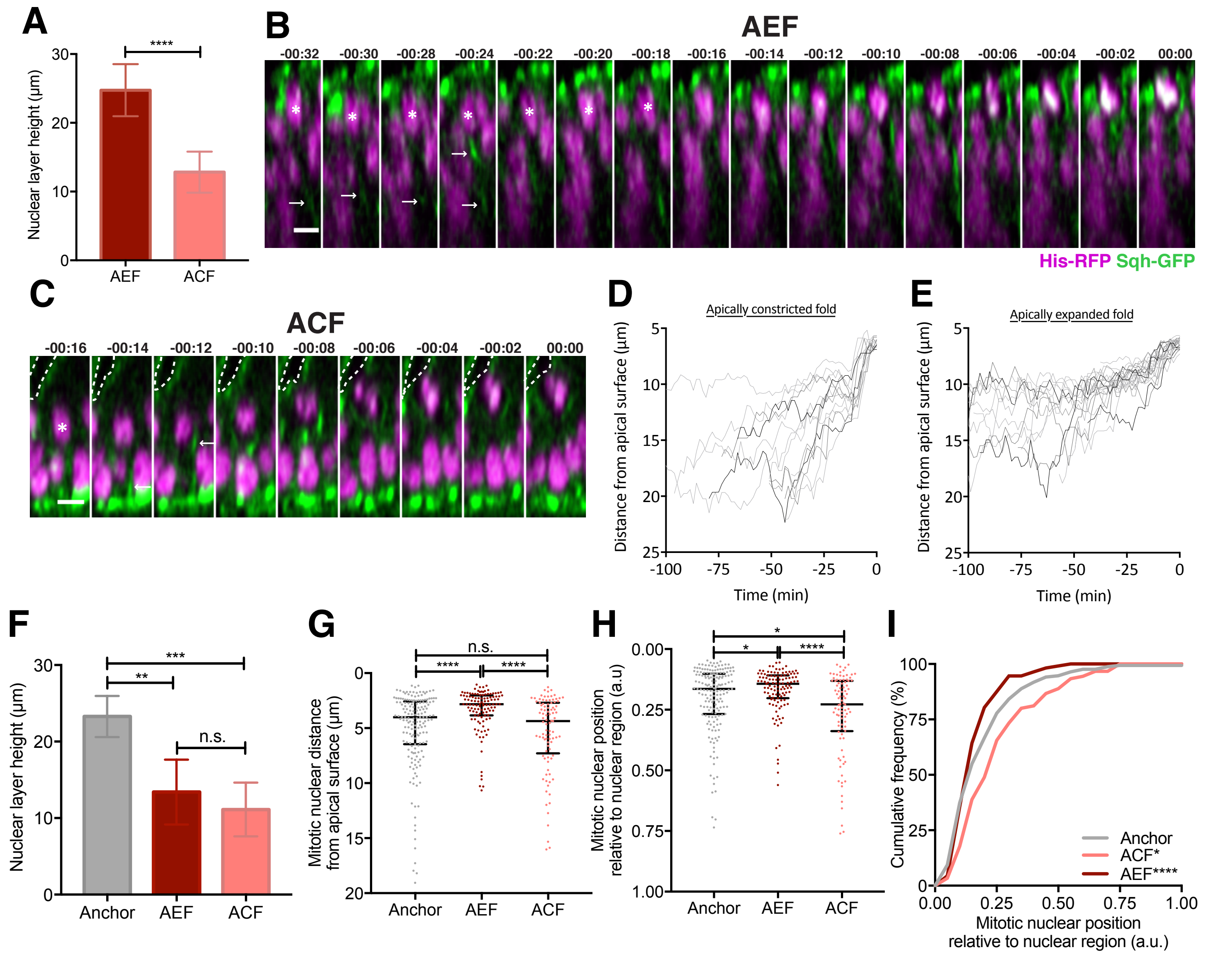


**Figure S4. Tissue folding influences mitotic nuclear positioning and dynamics. Related to Figure 4.**

(A) Nuclear layer height measurements from Figure 4B. (B, C) Representative time-lapses of mitotic nuclei moving apically in the distinct fold regions, the apically expanded fold (AEF) and apically constricted fold (ACF), in wing discs expressing Sqh-GFP and His-RFP. White arrows indicate cortical myosin enrichment. White asterisks mark the tracked nucleus when in crowded regions. White dashed line in C represents apical lumen region. (D, E) Individual nuclei tracks from the wing disc fold regions averaged in Figure 4C, D. (F) Nuclear layer height measurements from Figure 4H. (G) Mitotic nuclear distance from apical surface presented as dot plot, corresponding to Figure 4I. (H, I) Distance of mitotic nuclei from apical surface relative to nuclear region presented as dot plot (H) and cumulative frequency distribution (I). (A, D, E) n = 14 (AEF) and 20 (ACF) nuclear trajectories from 3 wing discs per condition. (F-I) n = 5 (anchored), 10 (AEF), 13 (ACF) wing discs. Scale bars: A,B, 5 $\mu$m. Statistical significance: A, unpaired t-test; F, one-way ANOVA; G-I, Kolmogorov-Smirnov comparison of cumulative distribution. *p<0.05, **p<0.01, ***p<0.001, ****p<0.0001. Error bars: A,D, mean ± SD; G-I, median ± interquartile range.

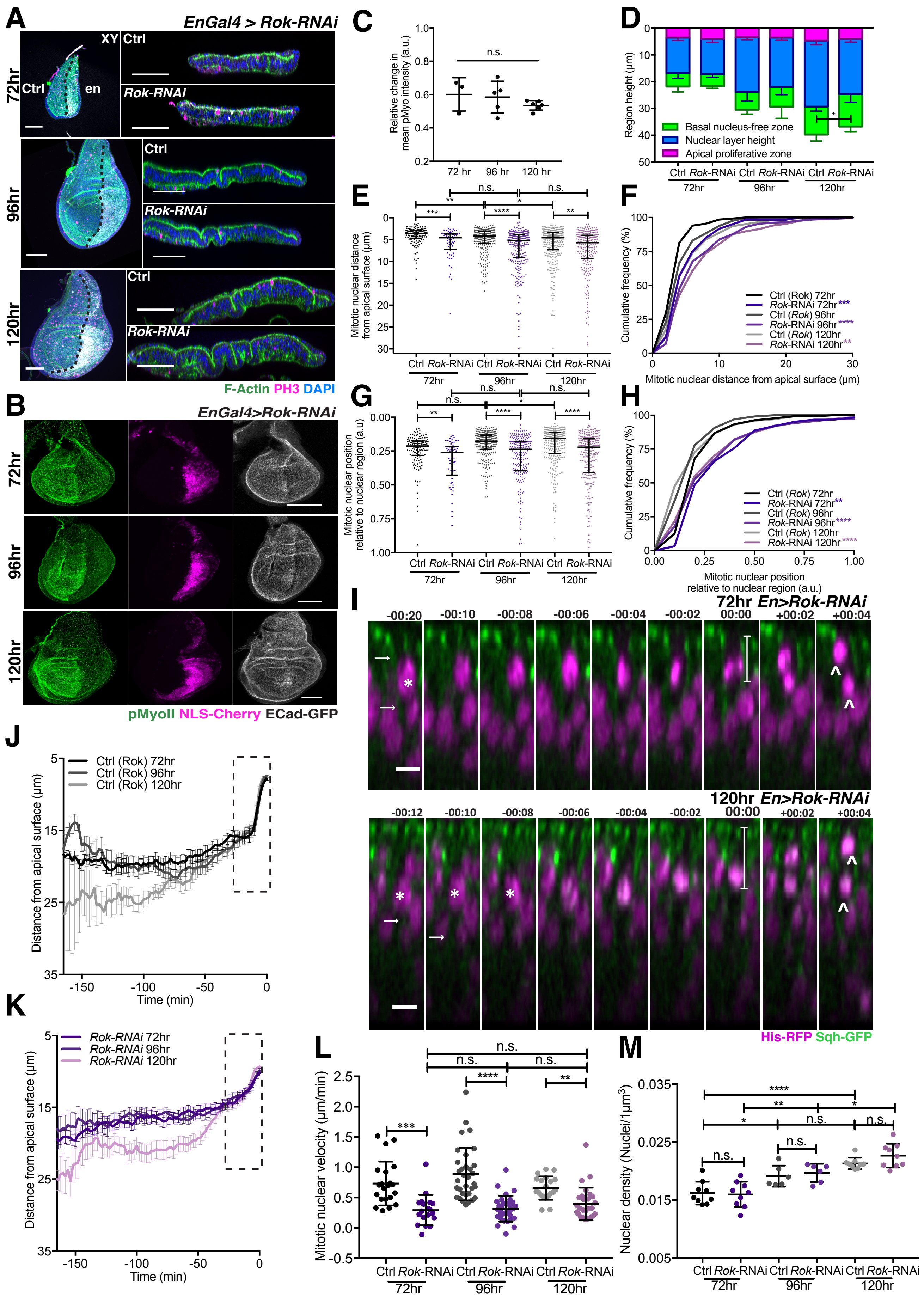

A
EnGal4 > Rok-RNAi
72hr
96hr
120hr
XY
Ctrl
en
Rok-RNAi
F-Actin PH3 DAPI
B
EnGal4>Rok-RNAi
pMyoII NLS-Cherry ECad-GFP
C
Relative change in mean pMyo intensity (a.u.)
n.s.
72 hr
96 hr
120 hr
D
Region height (µm)
Basal nucleus-free zone
Nuclear layer height
Apical proliferative zone
Ctrl Rok-RNAi Ctrl Rok-RNAi Ctrl Rok-RNAi
72hr 96hr 120hr
E
Mitotic nuclear distance from apical surface (µm)
F
Cumulative frequency (%)
Ctrl (Rok) 72hr
Rok-RNAi 72hr***
Ctrl (Rok) 96hr
Rok-RNAi 96hr****
Ctrl (Rok) 120hr
Rok-RNAi 120hr**
Mitotic nuclear distance from apical surface (µm)
G
Mitotic nuclear position relative to nuclear region (a.u)
H
Ctrl (Rok) 72hr
Rok-RNAi 72hr**
Ctrl (Rok) 96hr
Rok-RNAi 96hr****
Ctrl (Rok) 120hr
Rok-RNAi 120hr****
Mitotic nuclear position relative to nuclear region (a.u.)
I
72hr En>Rok-RNAi
-00:20 -00:10 -00:08 -00:06 -00:04 -00:02 00:00 +00:02 +00:04
120hr En>Rok-RNAi
-00:12 -00:10 -00:08 -00:06 -00:04 -00:02 00:00 +00:02 +00:04
His-RFP Sqh-GFP
J
Ctrl (Rok) 72hr
Ctrl (Rok) 96hr
Ctrl (Rok) 120hr
Distance from apical surface (µm)
Time (min)
K
Rok-RNAi 72hr
Rok-RNAi 96hr
Rok-RNAi 120hr
Distance from apical surface (µm)
Time (min)
L
Mitotic nuclear velocity (µm/min)
M
Nuclear density (Nuclei/1µm³)

**Figure S5. Rok is required for apical nuclear movement at all stages of development. Related to Figure 5.**

(A) Representative images of wing disc morphology with *Rok-RNAi* expression in *engrailed* domain (posterior, *en*) at different developmental stages. *Rok-RNAi* expression marked by UAS-NLS-Cherry in the posterior region (grey), phalloidin (green), anti-PH3 (magenta) and DAPI (blue). Black dashed line shows anterior-posterior boundary. (B) Representative images of phospho-Myosin-II (pMyo) staining in *EnGal4>Rok-RNAi* wing discs. Green staining shows anti-pMyoII, anterior region with UAS-NLS-Cherry in magenta and E-Cadherin-GFP in grey. (C) Relative change in pMyo staining mean intensity in *Rok-RNAi* region compared to control region. (D) Epithelial morphology measurements for wing discs in (E-H). Statistical difference between control and *Rok-RNAi* for only nuclear layer height at 120hr AEL. (E, F) Mitotic nuclear distance from apical surface for control and *Rok-RNAi* wing discs presented as dot plot (E) and cumulative frequency distribution (F). (G, H) Data in (E, F) normalised to nuclear region. (I) Representative images of *Rok-RNAi* mitotic nuclei moving apically within posterior pouch region at 72hr and 120hr AEL. Wing discs express Sqh-GFP, His-RFP and *EnGal4>UAS-Rok-RNAi*. Arrows: cortical myosin enrichment regions. Asterisks: tracked nuclei. Arrow heads: apico-basal cell division. Line bar: distance of metaphase nucleus from apical surface at 00:00 mins, with values presented in Figure 5I. (J, K) Full time-course for average nuclear tracks for anterior control (J) and posterior *Rok-RNAi* (K) mitotic cells, at each developmental stage. Black dashed line represents region enlarged in Figure 5B-E. (L) Mitotic nuclear velocity for control and *Rok-RNAi* expressing mitotic cells, at each developmental stage. Calculated from onset of apical movement and cortical mitotic enrichment, up to most-apical position, i.e. metaphase. For 72hr, 96hr and 120hr wildtype cells, the mean onset of apical movement was -11.1, -10.0 and -12.1 min respectively and the duration was 9.2, 8.0 and 9.4 min respectively. For 72hr, 96hr and 120hr *Rok-RNAi* expressing cells, the mean onset of apical movement was -9.9, -12.7 and -13.6 min respectively and the duration was 8.8, 11.8 and 12.1 min respectively. (M) Nuclear density per $\mu m^3$ for anterior control and posterior *Rok-RNAi* expressing wing disc regions, in Sqh-GFP and His-RFP backgrounds, at different developmental stages. Density significantly changes for *Rok-RNAi* region between time-points although wildtype region has delayed development. (B, C) n = 3 (72hr), 5 (96hr) and 6 (120hr) wing discs. (D-H) n = 8 (72hr), 7 (96hr) and 5 (120hr) wing discs. (J-M) n = 19-33 nuclear trajectories acquired from 3 wing discs per developmental stage. Scale bars: A, G, 50 $\mu$m; I, 5 $\mu$m. Statistical significance: E-H , Kolmogorov-Smirnov comparison of cumulative distribution; D, independent t-test; C, L and M, one-way ANOVA. n.s. $p>0.05$, *$p<0.05$, **$p<0.01$, ***$p<0.001$, ****$p<0.0001$. Error bars: E,G, median ± interquartile range; C, D, L, M, mean ± SD; J, K, mean ± SEM.

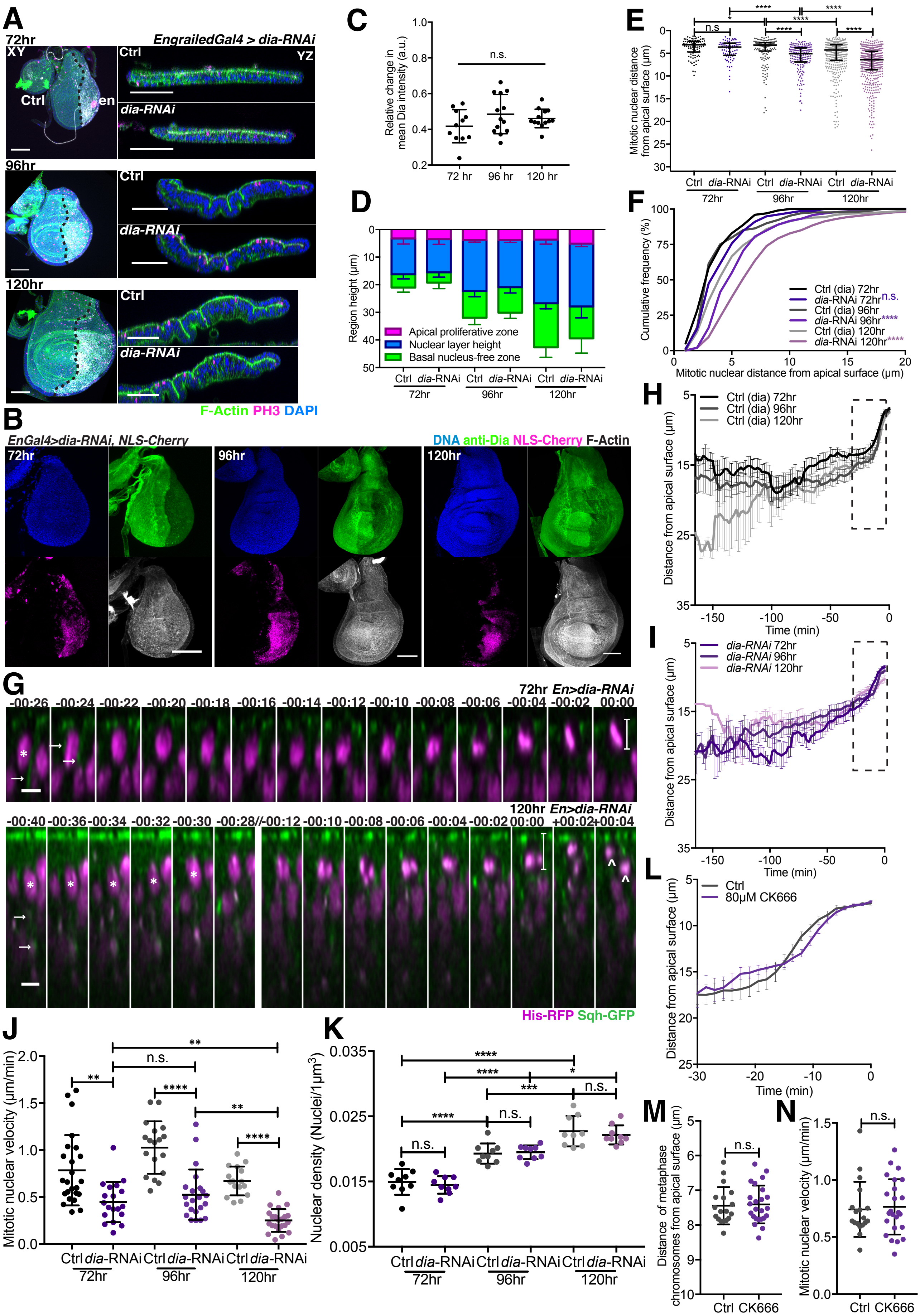

A
72hr
EngrailedGal4 > dia-RNAi
XY
YZ
Ctrl
en
dia-RNAi
96hr
120hr
F-Actin PH3 DAPI
B
EnGal4>dia-RNAi, NLS-Cherry
DNA anti-Dia NLS-Cherry F-Actin
C
Relative change in mean Dia intensity (a.u.)
n.s.
72 hr
96 hr
120 hr
D
Region height (µm)
Apical proliferative zone
Nuclear layer height
Basal nucleus-free zone
E
Mitotic nuclear distance from apical surface (µm)
F
Cumulative frequency (%)
Ctrl (dia) 72hr
dia-RNAi 72hr n.s.
Ctrl (dia) 96hr
dia-RNAi 96hr ****
Ctrl (dia) 120hr
dia-RNAi 120hr ****
Mitotic nuclear distance from apical surface (µm)
G
72hr En>dia-RNAi
120hr En>dia-RNAi
His-RFP Sqh-GFP
H
Distance from apical surface (µm)
Time (min)
I
dia-RNAi 72hr
dia-RNAi 96hr
dia-RNAi 120hr
J
Mitotic nuclear velocity (µm/min)
K
Nuclear density (Nuclei/1µm³)
L
Ctrl
80µM CK666
M
Distance of metaphase chromosomes from apical surface (µm)
N
Ctrl
CK666

**Figure S6. Dependency on Dia for apical mitotic nuclear movement increases with development. Related to Figure 6.**

(A) Representative images of wing disc morphology with *dia-RNAi* expression in *engrailed* domain (posterior, *en*) at different developmental stages. *dia-RNAi* expression marked by UAS-NLS-Cherry in the posterior region (grey), phalloidin (green), anti-PH3 (magenta) and DAPI (blue). Black dashed line shows anterior-posterior boundary. (B) Representative images of anti-Dia staining (green) in *EnGal4>dia-RNAi* wing discs with DAPI (blue), UAS-NLS-Cherry (magenta) and F-actin (grey). (C) Relative change in anti-Dia staining in *dia-RNAi* region compared to control region. (D) Epithelial morphology measurements for control and *dia-RNAi* wing discs at each developmental stage. (E, F) Mitotic nuclear distance from apical surface for control and *dia-RNAi* wing discs presented as dot plot (E) and cumulative frequency distribution (F). (G) Representative images of *dia-RNAi* mitotic nuclei moving apically within posterior pouch region at 72 and 120 hours AEL. Wing discs express Sqh-GFP, His-RFP and *EnGal4>UAS-dia-RNAi*. Arrows: cortical myosin enrichment regions. Asterisks: tracked nuclei. Arrow heads: apico-basal cell division. Line bar: distance of metaphase nucleus from apical surface at 00:00 mins, with values presented in Figure 6I. (H, I) Full time course for average nuclear tracks for anterior control (H) and posterior *dia-RNAi* (I) mitotic cells, at each developmental stage. Black dashed line represents region enlarged in Figure 6B-E. (J-K) Mitotic nuclear velocity, calculated from onset of apical movement and cortical mitotic enrichment, up to most-apical position, i.e. metaphase (J), and nuclear density per $\mu m^3$ (K) for anterior control and posterior *dia-RNAi* expressing wing disc regions in Sqh-GFP and His-RFP backgrounds, at different developmental stages. For calculating the mitotic nuclear velocity, for 72hr, 96hr and 120hr wildtype cells, the mean onset of apical movement was -10.2, -9.8 and -9.3 min respectively and the duration was 7.1, 5.8 and 7.5 min respectively. For 72hr, 96hr and 120hr *dia-RNAi* expressing cells, the mean onset of apical movement was –13.7, -9.9 and -9.9 min respectively and the duration was 9.6, 7.7 and 8.7 min respectively. (C) n = 11 (72hr), 13 (96hr), 13 (120hr) wing discs. (D-F) n = 4 (72hr), 7 (96hr) and 8 (120hr) wing discs. (H-K) n = 16-24 nuclear trajectories acquired from 3-4 wing discs per developmental stage. Scale bars: A, E, 50$\mu$m, G, 5$\mu$m. Statistical significance: C, J, K; one-way ANOVA; E, Kolmogorov-Smirnov comparison of cumulative distribution. n.s. $p>0.05$, *$p<0.05$, **$p<0.01$, ****$p<0.0001$. Error bars: C, J, K, mean ± SD; E, median ± interquartile range; H, I, mean ± SEM.

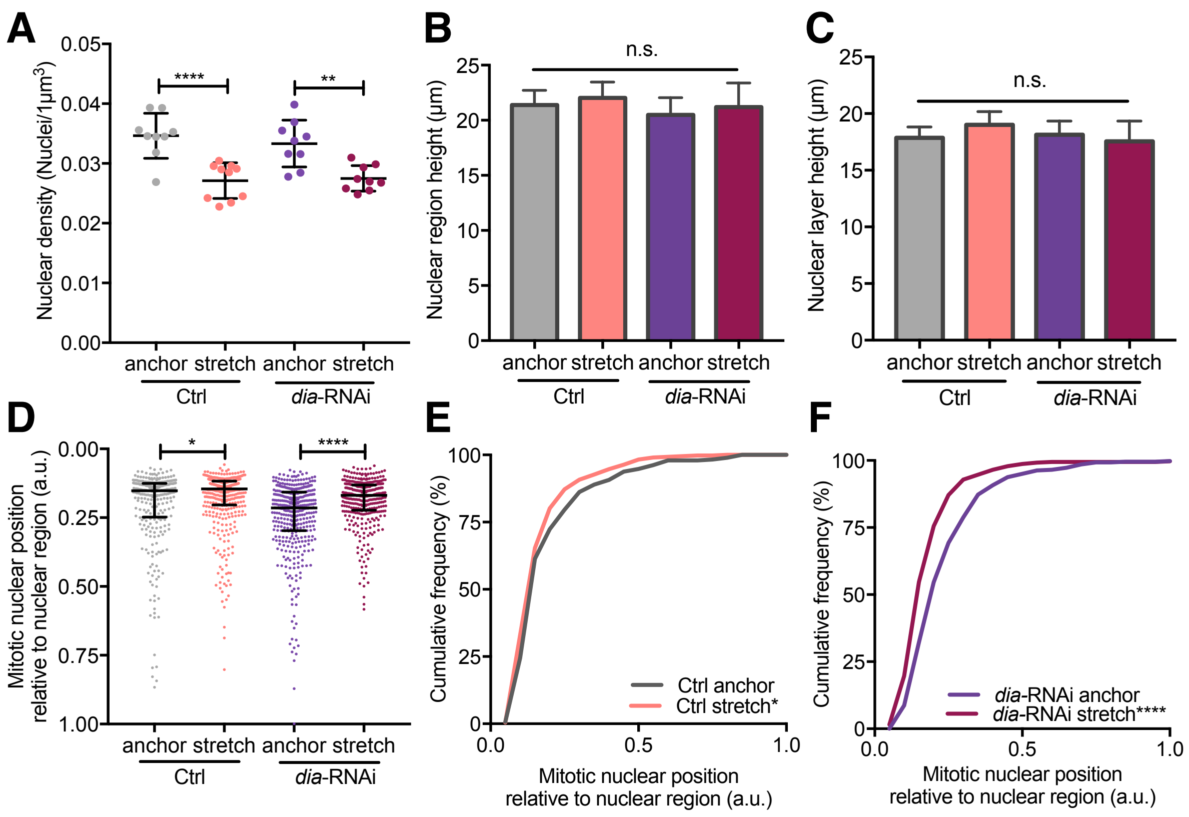


**Figure S7. Defects in nuclear positioning upon Dia depletion can be rescued by mechanically reducing cell density. Related to Figure 7.**

(A-C) Density of nuclei per 1$\mu m^3$ (A), nuclear region height (B), and nuclear layer height (C), in pouch region for control and *dia-RNAi* expressing discs in anchor and stretched conditions. (D-E) Mitotic nuclear position relative to the nuclear region for control (E) and *dia-RNAi* expressing (F) wing disc pouch regions in anchor and stretched conditions presented as dot plot (D) and cumulative frequency distribution (E, F). Data corresponds to Figure 7B-D. (A) n = 3 wing discs per condition. (B-F) n = 7 (anchored) and 9 (stretched) control wing discs, and n = 9 (anchored) and 8 (stretched) *dia-RNAi* wing discs. Statistical significance: A, unpaired t-test; B, C, one-way ANOVA; D-F, Kolmogorov-Smirnov comparison of cumulative. n.s. $p>0.05$, *$p<0.05$, **$p<0.01$, ****$p<0.0001$. Error bars: A-C, mean ± SD; D, median interquartile range.
