# Supplementary material for: Tissue Mechanics Regulate Mitotic Nuclear Dynamics during Epithelial Development
Source: Curr Biol. 2020 Jul 6;30(13):2419–2432.e4. doi: 10.1016/j.cub.2020.04.041 (PMC7342018; doi:10.1016/j.cub.2020.04.041)
Supplement: Document S2. Article plus Supplemental Information [file mmc2.pdf]

Current Biology

Article

Tissue Mechanics Regulate Mitotic Nuclear Dynamics during Epithelial Development

Graphical Abstract

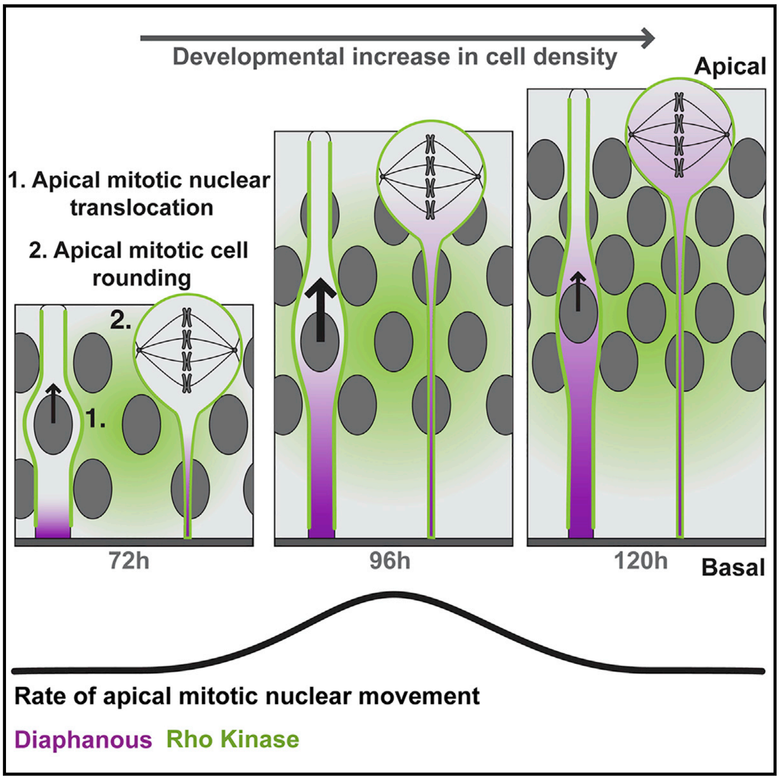

Highlights

- Mitotic nuclear dynamics change as the *Drosophila* wing disc develops and grows
- Cell density is the primary driver of the differences in mitotic nuclear dynamics
- Mitotic rounding and nuclear dynamics depend on Dia in a density-dependent manner
- Nuclear dynamic defects in Dia mutants can be reversed by physical perturbations

Authors

Natalie J. Kirkland, Alice C. Yuen, Melda Tozluoglu, Nancy Hui, Ewa K. Paluch, Yanlan Mao

Correspondence

ekp25@cam.ac.uk (E.K.P.), y.mao@ucl.ac.uk (Y.M.)

In Brief

By using genetic and physical manipulations of cell packing, Kirkland et al. find a density-dependent regulation of mitotic nuclear dynamics in the developing *Drosophila* wing disc. Moreover, they show a differential requirement of Rok and Dia for mitotic rounding and apical nuclear positioning as cell density increases during development.

Kirkland et al., 2020, Current Biology *30*, 2419–2432
July 6, 2020 © 2020 The Authors. Published by Elsevier Inc.
https://doi.org/10.1016/j.cub.2020.04.041

CellPress

Current Biology

CellPress
OPEN ACCESS

Article

Tissue Mechanics Regulate Mitotic Nuclear Dynamics during Epithelial Development

Natalie J. Kirkland,[1] Alice C. Yuen,[1,2] Melda Tozluoglu,[1] Nancy Hui,[1] Ewa K. Paluch,[1,3,4,*] and Yanlan Mao[1,3,5,6,*]
[1]MRC Laboratory for Molecular Cell Biology, University College London, Gower Street, London WC1E 6BT, UK
[2]Department of Cell and Developmental Biology, University College London, Gower Street, London WC1E 6BT, UK
[3]Institute for the Physics of Living Systems, University College London, Gower Street, London WC1E 6BT, UK
[4]Department of Physiology, Development and Neuroscience, University of Cambridge, Downing Street, Cambridge CB2 3DY, UK
[5]Twitter: @YanlanMao
[6]Lead Contact
*Correspondence: ekp25@cam.ac.uk (E.K.P.), y.mao@ucl.ac.uk (Y.M.)
https://doi.org/10.1016/j.cub.2020.04.041

SUMMARY

Cell divisions are essential for tissue growth. In pseudostratified epithelia, where nuclei are staggered across the tissue, each nucleus migrates apically before undergoing mitosis. Successful apical nuclear migration is critical for planar-orientated cell divisions in densely packed epithelia. Most previous investigations have focused on the local cellular mechanisms controlling nuclear migration. Inter-species and inter-organ comparisons of different pseudostratified epithelia suggest global tissue architecture may influence nuclear dynamics, but the underlying mechanisms remain elusive. Here, we use the developing *Drosophila* wing disc to systematically investigate, in a single epithelial type, how changes in tissue architecture during growth influence mitotic nuclear migration. We observe distinct nuclear dynamics at discrete developmental stages, as epithelial morphology changes. We use genetic and physical perturbations to show a direct effect of cell density on mitotic nuclear positioning. We find Rho kinase and Diaphanous, which facilitate mitotic cell rounding in confined cell conditions, are essential for efficient apical nuclear movement. Perturbation of Diaphanous causes increasing defects in apical nuclear migration as the tissue grows and cell density increases, and these defects can be reversed by acute physical reduction of cell density. Our findings reveal how the mechanical environment imposed on cells within a tissue alters the molecular and cellular mechanisms adopted by single cells for mitosis.

INTRODUCTION

Successful mitosis in densely packed epithelia relies on the ability of cells to round up. Rounding is driven by reorganization of cellular actin into a highly contractile actomyosin cortex at the cell membrane [1–6] and generates the physical space required for mitotic spindle assembly and chromosome separation [2, 7]. Indeed, mitotic rounding is required to resist the mechanical constraints exerted by neighboring cells, and when defective can lead to aneuploidy and division failure [3, 8]. In most epithelia, mitotic cells round up at the apical surface of the tissue, facilitating planar-orientated cell divisions [9, 10].

Pseudostratified epithelia (PSE), where nuclei are staggered across an epithelial monolayer, are among the most densely packed tissue structures. PSE are found in abundant and diverse developing organs, from the intestine [11] to the neocortex [12]. Accordingly, PSE architecture can differ drastically, varying in height, curvature, and nuclear density [13]. For division to occur, nuclei must translocate to the apical surface prior to mitosis, a process referred to as interkinetic nuclear migration (IKNM) [14].

IKNM studies have revealed a variety of mechanisms driving nuclear movement [13, 15, 16]. Tall neuroepithelia found in the developing mammalian cortex utilize microtubule-dependent mechanisms [17–21], while shorter zebrafish neuroepithelia utilize actomyosin-dependent mechanisms [22–25]. In both cases, apical nuclear movement occurs in the G2 phase of the cell cycle, prior to mitotic rounding [18–20, 22, 23]. However, *Drosophila* PSE, including the tall wing disc epithelia [24] and short neuroepithelia [25], appear to drive nuclear movement coincident with mitotic rounding, a timing conserved with other species, including the sea anemone, *Nemostella vectensis* [24].

Although previous studies have mostly focused on the processes controlling IKNM within cells, comparative studies suggest that IKNM dynamics might also be influenced by tissue architecture [26–28]. Differing mechanical constraints exerted by epithelial morphology may result in nuclear movement that is driven by distinct molecular machinery [28]. To what extent and how tissue properties influence nuclear dynamics remain unclear, as currently only epithelia from different species or organs have been compared [26–28].

Here, we use the *Drosophila* wing disc to investigate the effect of tissue architecture on IKNM. The wing disc is comprised largely of a pseudostratified, columnar epithelium that increases in size, and changes in shape, during larval development. We find distinct mitotic nuclear dynamics as the wing disc mechanical properties change. Using genetic and mechanical

This is an open access article under the CC BY license (http://creativecommons.org/licenses/by/4.0/).

perturbations, we show that cell density mediates differences in mitotic nuclear behavior. Finally, we show that while Rho kinase (Rok) is indispensable for efficient apical mitotic positioning, dependency on Diaphanous (Dia) increases as cell density increases. Our findings reveal how the mechanical environment imposed on cells within tissues can influence the molecular mechanisms used to ensure robust apical mitosis.

RESULTS

Wing Disc Development Is Associated with Increased Tissue Height, Nuclear Layering, and Cell Density

To identify features of cell and tissue morphology that may influence mitotic nuclear behavior, we first characterized how the apico-basal architecture of the wing disc changes during development. We used wing discs at 72, 96, and 120 hours (h) after egg laying (AEL) as they exhibit distinct tissue morphologies, differing in size and three-dimensional architecture (Figure 1A). We found that tissue height progressively increased in the pouch region (Figures 1A, 1C, and S1B) and accompanied changes in apico-basal nuclear organization. We measured three zones along this axis, the apical proliferative zone, the nuclear layer (NL), and the basal nucleus-free zone, and observed striking lengthening of the NL during development (Figures 1B, 1C, and S1A–S1E). The average number of nuclei stacked within the NL and their density also increased (Figures 1D and 1E), suggesting these changes help accommodate increasing cell numbers during development.

Developmental Changes in Tissue Architecture Are Associated with Distinct Patterns of Mitotic Nuclear Positioning

We then asked whether mitotic nuclear behavior changed with changing wing disc architecture. We used anti-phospho-histone H3 (PH3) to mark mitotic nuclei and measured their distance to the apical surface, at each developmental stage (Figure 1F). We found that as development progressed, more PH3+ nuclei were distributed further from the apical surface (Figures 1G and 1H). To specifically assess the nuclei translocating to the apical surface, we analyzed the proportion of mitotic nuclei positioned more than 5 μm from the apical surface per wing disc, and found that it increased through development (Figure 1I). When normalized to account for the potential distance they must translocate to the apical surface at each stage (the "nuclear region"; Figure S1F), we found that mitotic nuclei were most apically distributed at 96 h AEL compared to wing discs at 72 and 120 h AEL (Figures S1G and S1H). Our findings suggest that differences in wing disc morphology accompanying development influence mitotic nuclei behavior.

Mitotic Nuclear Dynamics Depend on Tissue Architecture

The broader distribution of mitotic nuclei from the apical surface as development progresses indicates differences in mitotic nuclear dynamics. Therefore, we assessed mitotic nuclear movement using live imaging of developing wing discs expressing the regulatory light chain of non-muscle myosin-II tagged with GFP (Sqh-GFP) and histone 2A-RFP (His-RFP). Individual and averaged nuclear trajectories showed nuclei displayed little persistence until a sharp transition to apically directed movement, occurring shortly before metaphase (Figures 2A, 2B, and S2A–S2C). Moreover, for each developmental stage, the instantaneous velocity of the nuclear trajectories initially fluctuated around 0, followed by a persistent increase (Figures 2C–2E).

Analyzing 72 h AEL wing discs, where the lateral cell membranes can be most easily distinguished, we found the transition to apical, directed movement correlated with enrichment of Sqh-GFP at the cell cortex (Figures 2A and 2I). As myosin is a key driver of mitotic rounding, this suggests that the transition to persistent apical motion may represent the onset of mitotic rounding.

To quantitatively assess whether the distinct phases in the nuclear trajectories represent distinct modes of nuclear motion, we carried out mean square displacement (MSD) analysis on the individual trajectories at each developmental stage. First, we divided the trajectories into "early" and "late" phases as described in the STAR Methods (Figures 2C–2E). We then calculated the MSD/2D versus time lag of nuclei for each phase (where D is a fitted diffusion constant; STAR Methods; Figures 2F–2H). The slope of a linear fit to the MSD/2D in a log-log representation gives a readout of the type of movement displayed, with a slope of 1.0 corresponding to stochastic diffusion, values above 1.0 indicating directional movement, and a slope of 2.0 indicating ballistic movement (for details, see STAR Methods). We found at each developmental stage that the change in the fitted slopes between the early and late phases indicated a transition from diffusive motion (or sub-diffusive at 72 h) toward more directed motion. Together with our analysis of Sqh-GFP intensities, our observations suggest that apical nuclear motion becomes more directed at late phases, concomitant with mitotic rounding.

We next asked whether differences in nuclear dynamics between developmental stages could explain the differences in mitotic positioning in fixed tissues (Figures 1G–1I). Examining the late-phase instantaneous nuclear velocity and the average mitotic nuclear velocity (calculated from the onset of upward movement to metaphase), we found that mitotic nuclear velocity was greatest at 96 h AEL, followed by 120 h AEL, then 72 h AEL (Figures 2C–2E and 2J), consistent with our fixed-tissue analysis. Our results therefore indicate that development is associated with differences in nuclear dynamics.

Perturbing Cell Density Influences Mitotic Nuclear Positioning

We hypothesized that the increasing cell density (Figure 1E) may regulate nuclear velocity between developmental stages. Therefore, we sought to perturb cell density and quantify nuclear position in fixed tissues. We first genetically altered cell density by perturbing Perlecan (Trol), a component of the extracellular matrix, which, without affecting the number of cells, can decrease and increase wing disc surface area when reduced or overexpressed, respectively [29, 30] (Figure 3A). Wing discs expressing *trol-RNAi* showed an increased epithelial height, NL height, and nuclear density (Figures 3B, 3C, S3A, and S3B), and a corresponding shift in the distribution of PH3+ nuclei away from the apical surface (Figures 3D, 3E, and S3C–S3E). Conversely, increasing Perlecan/*trol* decreased epithelial height, NL height, and nuclear density (Figures 3F, 3G, S3F, and S3G), and shifted PH3+ nuclei toward the apical surface (Figures 3H,

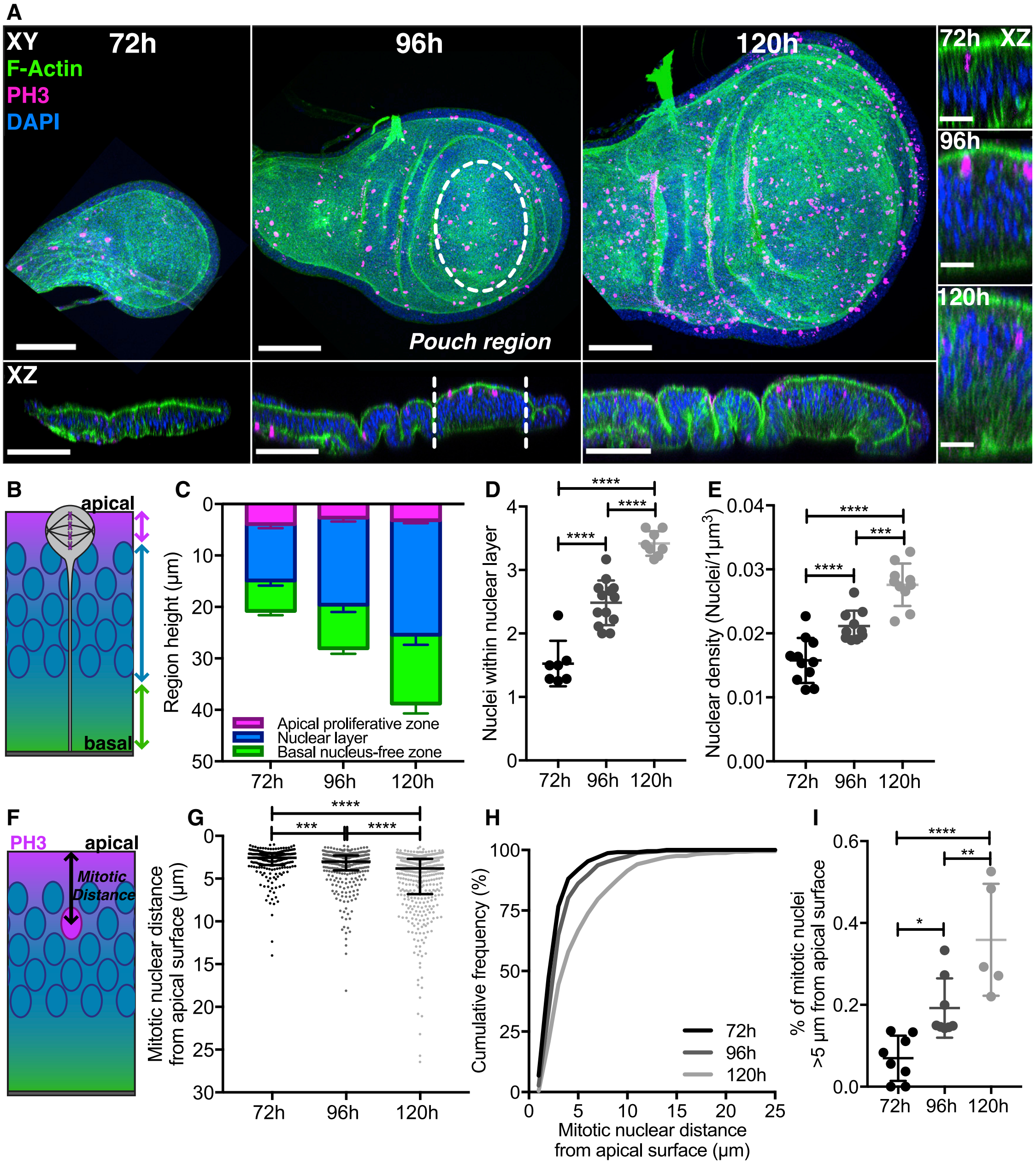

Figure 1. Changes in the Wing Disc Architecture during Development Are Associated with Distinct Patterns of Mitotic Nuclear Positioning

(A) *Drosophila* wing discs at 72, 96, and 120 h AEL, fixed and stained with phalloidin (green), anti-PH3 (magenta), and DAPI (blue). Top left: projection. Bottom left: cross-sections. Analyzed region highlighted with white dashed lines. Right: enlarged cross-sections. Scale bars, 50 μm (left) and 10 μm (right).

(B) Schematic highlighting sub-regions along the apico-basal axis, quantified in (C). Magenta, apical proliferative zone; blue, nuclear layer; green, basal nucleus-free zone.

(C) Apico-basal sub-region height at developmental stages, quantified as in Figure S1A. Individual sub-regions in Figures S1B–S1E. n = 8 (72 h), 8 (96 h), and 5 (120 h) wing discs.

(D) Average number of nuclei within the nuclear layer region per disc, quantified as in Figure S1A. n = 7 (72 h), 13 (96 h), and 8 (120 h) wing discs.

(E) Density of nuclei per 1 μm^3. n = 3 wing discs per stage.

(F) Schematic for mitotic nuclear distance, the distance of PH3+ nuclei from the apical surface.

(G and H) Mitotic nuclear distance presented as dot plot in (G) and cumulative frequency distribution in (H). Normalized data shown in Figures S1F–S1H.

(I) Percentage of mitotic nuclei found more than 5 μm from apical surface. n = 8 (72 h), 8 (96 h), and 5 (120 h) wing discs.

Statistical significance: (D), (E), and (I), one-way ANOVA; (G), Kolmogorov-Smirnov comparison of cumulative distribution. *$p < 0.05$, **$p < 0.01$, ***$p < 0.001$, ****$p < 0.0001$. Error bars: (C)–(E) and (I), mean ± SD; (G), median ± interquartile range.

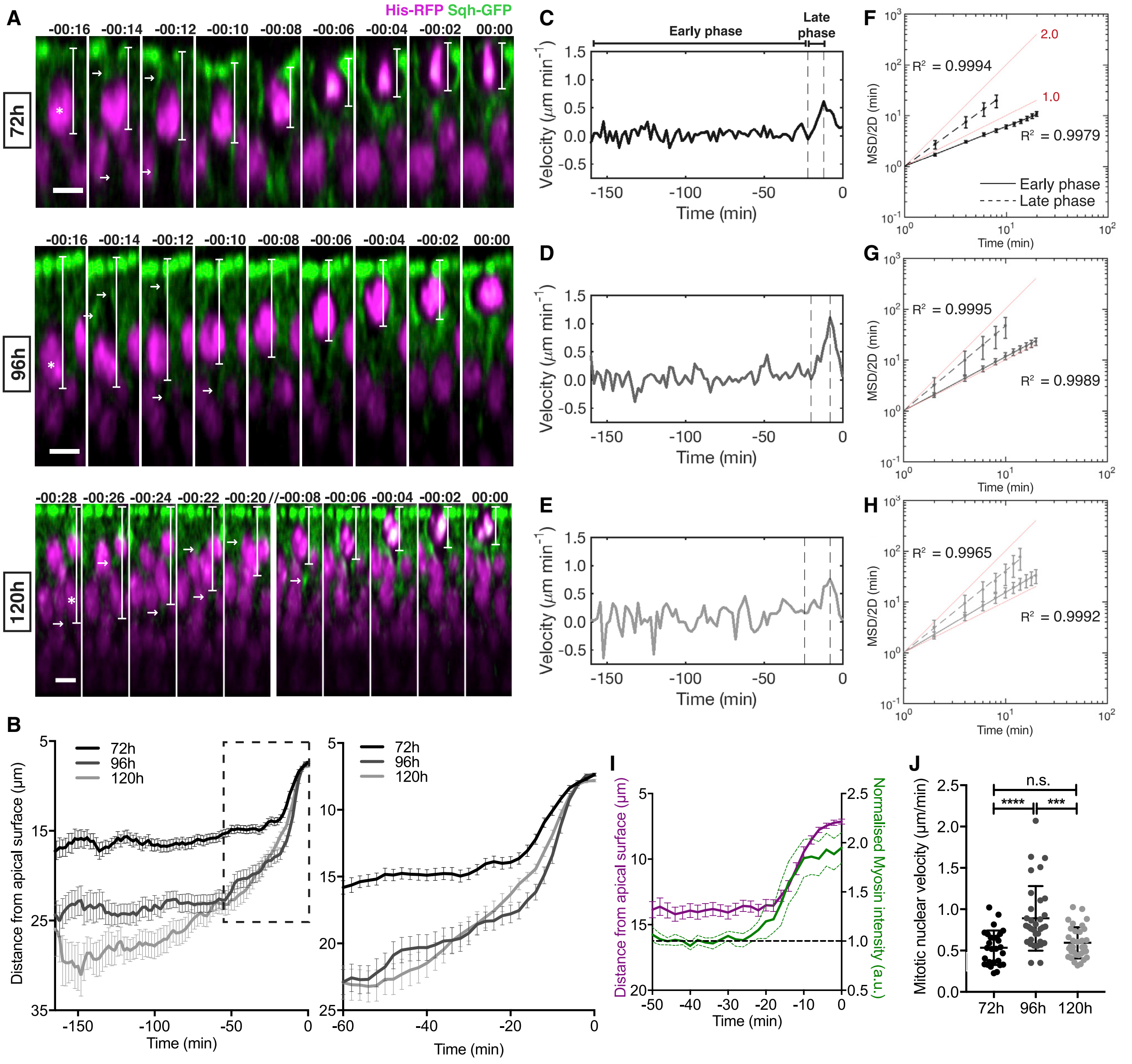

Figure 2. Mitotic Nuclear Dynamics Depend on Tissue Architecture

(A) Time-lapse images of mitotic nuclear movement for Sqh-GFP, His-RFP wing discs at 72, 96, and 120 h AEL. White lines: measurement of nuclei movement for analysis in (B). Arrows: cortical Sqh-GFP enrichment. Double slash: skipped time steps. 00:00 min represents final metaphase. Scale bars, 5 µm.

(B) Average mitotic nuclei track at 72, 96, and 120 h AEL. Inset: transition to directed apical movement. Figures S2A–S2C show individual tracks.

(C–E) Average instantaneous nuclear velocity (C, 72 h; D, 96 h; E, 120 h AEL). Early phase, non-directed movement; late phase, directed nuclear movement.

(F–H) Log-log plot of MSD/2D versus time lag (F, 72 h; G, 96 h; H, 120 h AEL). Solid line, early phase; dashed line, late phase. Slopes for early and late phases, respectively: 72 h, 0.78 and 1.45; 96 h, 1.06 and 1.66; 120 h, 1.18 and 1.61. R^2 values are displayed in each panel. Red lines show lines of slope 1 (corresponding to diffusive movement) and slope 2 (corresponding to ballistic movement).

(B–H) n = 28, 33, and 34 nuclear trajectories from 3 (72 h), 3 (96 h), and 4 (120 h) wing discs, respectively.

(I) Normalized lateral membrane Sqh-GFP intensity (green) and nucleus distance from apical surface (magenta) for 72 h discs. n = 16 mitotic cells from 3 wing discs.

(J) Mitotic nuclear velocity for each track calculated from onset of apical movement and cortical Sqh-GFP enrichment, to most-apical metaphase. For 72, 96, and 120 h discs, mean onset of upward movement was −15.4, −13.8, and −18.5 min, and duration of upward movement was 11.1, 10.7, and 15.7 min, respectively. n = 28, 33, and 34 nuclear trajectories from 3 (72 h), 3 (96 h), and 4 (120 h) wing discs, respectively.

Statistical significance: (J), one-way ANOVA. n.s., $p > 0.05$; ***$p < 0.001$, ****$p < 0.0001$. Error bars: (B) and (I) (magenta), mean ± SEM; (F)–(I) (green), mean ± 95% confidence intervals; (J), mean ± SD.

CellPress
OPEN ACCESS

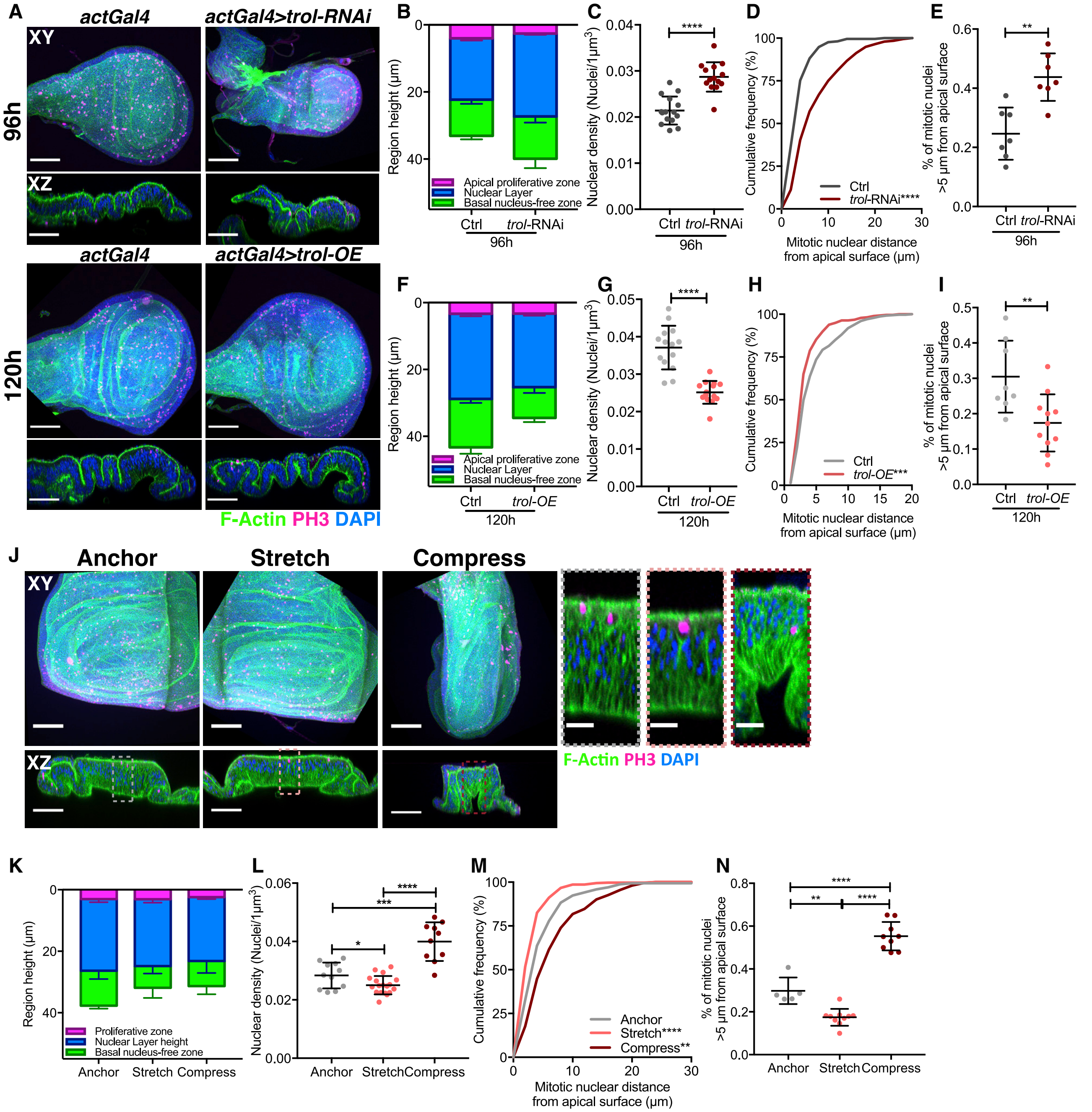

Figure 3. Perturbing Cell Density Influences Mitotic Nuclear Positioning

(A) Wing discs stained with phalloidin (green), anti-PH3 (magenta), and DAPI (blue). Top panel, left: 96 h *actin*Gal4 control, right: *UAS-trol*-RNAi (*perlecan*) driven by *actin*Gal4 at 96 h. Lower panel, left: 120 h *actin*Gal4 control, right: *UAS-trol* driven by *actin*Gal4 at 120 h. Top view, projections; lower view, cross-sections.

(B and F) Epithelial apico-basal morphology quantification for *trol*-RNAi (B), *trol-OE* (F), and respective controls. n = 7 wing discs per condition (B). n = 8 (control) and 11 (*trol-OE*) (F).

(C and G) Nuclear density per μm^3 for *trol*-RNAi (C), *trol-OE* (G), and respective controls. n = 3 wing discs per condition.

(D and H) Cumulative frequency distribution of mitotic nuclear distance, corresponding dot plots, and normalized values in Figures S3D–S3F and S3J–S3L for *trol*-RNAi (D), *trol-OE* (H), and respective controls. n = 7 wing discs per condition (D). n = 8 (control) and 11 (*trol-OE*) (H).

(E and I) Percentage of mitotic nuclei more than 5 µm from apical surface for *trol*-RNAi (E), *trol-OE* (I), and respective controls. n = 7 wing discs per condition (E). n = 8 (control) and 11 (*trol-OE*) (I).

(J) Wild-type wing discs 120 h AEL following 30-min mechanical perturbation, stained as in (A). Anchor: disc within device without stretch/compression. Right inset: enlarged view of cross-sections highlighting cell height and nuclear density changes.

(K) Epithelial apico-basal morphology for anchored, stretched, and compressed conditions. Corresponding data in Figures S3M–S3O.

(L) Nuclear density for anchored, stretched, and compressed conditions. n = 3 wing discs per condition.

(legend continued on next page)

3I, and S3H–S3J). This supports a role for cell density in regulating mitotic nuclear behavior; however, it does not exclude an influence of the NL height, which was considerably affected, and other confounding factors resulting from changes in extracellular matrix.

To acutely change cell density, while minimally affecting the NL height, we used a mechanical device [31] to stretch and compress the tissue (Figure 3J). Wild-type discs were cultured *ex vivo* in anchored (unperturbed), stretched, or compressed conditions (executed in <10 s, sustained for 30 min; Figure 3J). We found that while the height of the wing disc was altered upon stretch and compression, the nuclear region and the NL heights were not significantly affected compared to anchored controls (Figures 3J, 3K, and S3K–S3M). We previously demonstrated that wing disc remodeling upon stretch takes over 30 min [31]. Thus, here, nuclei are unlikely to extensively rearrange their position in the NL. As a result, nuclear density decreased and increased upon stretching and compression, respectively, while NL height remained unchanged (Figure 3L). We found stretching shifted mitotic nuclei toward the apical surface, while compression shifted mitotic nuclei away from the apical surface (Figures 3M, 3N, and S3N). Stretching and compressing could invoke additional effects on the tissue, including altering tension [31]. Nonetheless, our findings further support a role for cell density in regulating mitotic positioning and nuclear dynamics.

Tissue Folding Influences Mitotic Nuclear Positioning and Dynamics

To further explore how tissue architecture affects nuclear dynamics, we exploited the natural folds that form in the wing disc at later developmental stages. The wing disc possesses distinct folds with opposing curvatures: an apically expanded fold (AEF) and an apically constricted fold (ACF) (Figure 4A), which show different apico-basal distributions of nuclei (Figures 4B and S4A). To address how folds influence nuclear dynamics, we live-imaged the AEF and ACF in discs at 120 h AEL. We found striking differences in nuclear dynamics (Figures 4C–4E and S4B–S4E). Apical mitotic nuclear velocity was significantly greater for the ACF than the AEF (Figure 4E). As we had expected the increased apical surface area of the AEF to be favorable to mitotic nuclear movement, we quantified nuclear density to ask whether this instead mediated the reduced apical movement. Accordingly, nuclear density was greater in the AEF compared to the ACF (Figure 4F), suggesting that nuclear density has a greater impact on mitotic nuclear dynamics in folded regions.

To directly test if curvature influences mitotic nuclei behavior, we used our tissue manipulator device to acutely induce folds in the 120 h wing disc pouch (Figure 4G). Interestingly, in contrast to natural folds, mechanically induced folds displayed comparable nuclear layering in AEF and ACF, allowing us to assess the effect of curvature independently of changes in nuclear density (Figures 4H and S4F). We found that in the positive-curvature AEF, mitotic nuclei were distributed closer to the apical surface compared to the negative-curvature ACF (Figures 4I, 4J, and S4G–S4I). This suggests that curvature can influence mitotic positioning, but in the wing disc, cell density dominates mitotic nuclear behavior.

Rok Is Required for Apical Mitotic Nuclear Movement at All Stages of Development

So far, we have shown that mitotic nuclear dynamics depend on the mechanical properties of the wing disc, predominantly cell density. We therefore asked whether the molecular machinery driving nuclear movement also changes as tissue properties change. We first explored the role of Rok, a known effector of mitotic nuclear movement [22–24], which is also required to generate force for mitotic rounding in confined environments [3, 4, 8, 10]. We expressed *Rok-RNAi* using *engrailed-Gal4*, which drives expression in half the wing disc, preserving the other half as an age-matched control, and assessed mitotic positioning during development (Figures S5A–S5D). In Rok-depleted epithelia, mitotic nuclei were distributed further from the apical surface (Figures S5E and S5F), consistent with previous work [24]. Moreover, mitotic nuclei were shifted by a similar degree at each developmental stage, suggesting that Rok is equally important throughout development (Figures S5G and S5H).

To gain further insight, we assessed nuclear dynamics. We observed a consistent defect in nuclear trajectories, late-phase instantaneous nuclear velocity, and average mitotic nuclear velocity with *Rok-RNAi* (Figures 5A–5H and S5I–S5L). Similar to our fixed sample analysis, between developmental stages we found a comparable relative reduction in mitotic nuclear velocity (Figure 5I). Although expressing *Rok-RNAi* appeared to globally delay wing disc development, we confirmed that nuclear density increased incrementally within the *Rok-RNAi* compartment (Figure S5M).

In addition to affecting nuclear velocity, depleting Rok produced a consistent defect in apical positioning and a significant reduction in mitotic rounding across all developmental stages (Figures 5J and 5K). Consequently, *Rok-RNAi* cells often divided along the apico-basal axis rather than orientating along the planar axis (Figures 5A and S5I, white arrow heads), supporting the importance of Rok and mitotic rounding for planar-oriented division [9, 10]. Together, these results show that Rok is essential for efficient apical mitotic nuclear movement, independent of epithelial architecture.

To shed light on the mechanism by which Rok mediates its effect on apical mitotic positioning, we generated small clones expressing *Rok-RNAi* (Figure 5L), thereby allowing us to assess whether wild-type neighbors could rescue *Rok-RNAi* defects. *Rok-RNAi*-expressing cells neighboring wild-type cells exhibited

(M) Cumulative frequency distribution of mitotic nuclear distance for anchored, stretched, and compressed conditions with corresponding scatterplot in Figure S3P.

(N) Percentage of mitotic nuclei more than 5 μm from apical surface for anchored, stretched, and compressed conditions.

(K, M, and N) n = 3 (anchored), 8 (stretched), and 6 (compressed) wing discs.

Scale bars, 50 μm (A and J, left) and 10 μm (J, right). Statistical significance: (C), (E), (G), and (I), unpaired t test; (D), (H), and (M), Kolmogorov-Smirnov comparison of cumulative distribution; (L) and (N), one-way ANOVA. *$p < 0.05$, **$p < 0.01$, ***$p < 0.001$, ****$p < 0.0001$. Error bars: mean ± SD in all plots.

CellPress
OPEN ACCESS

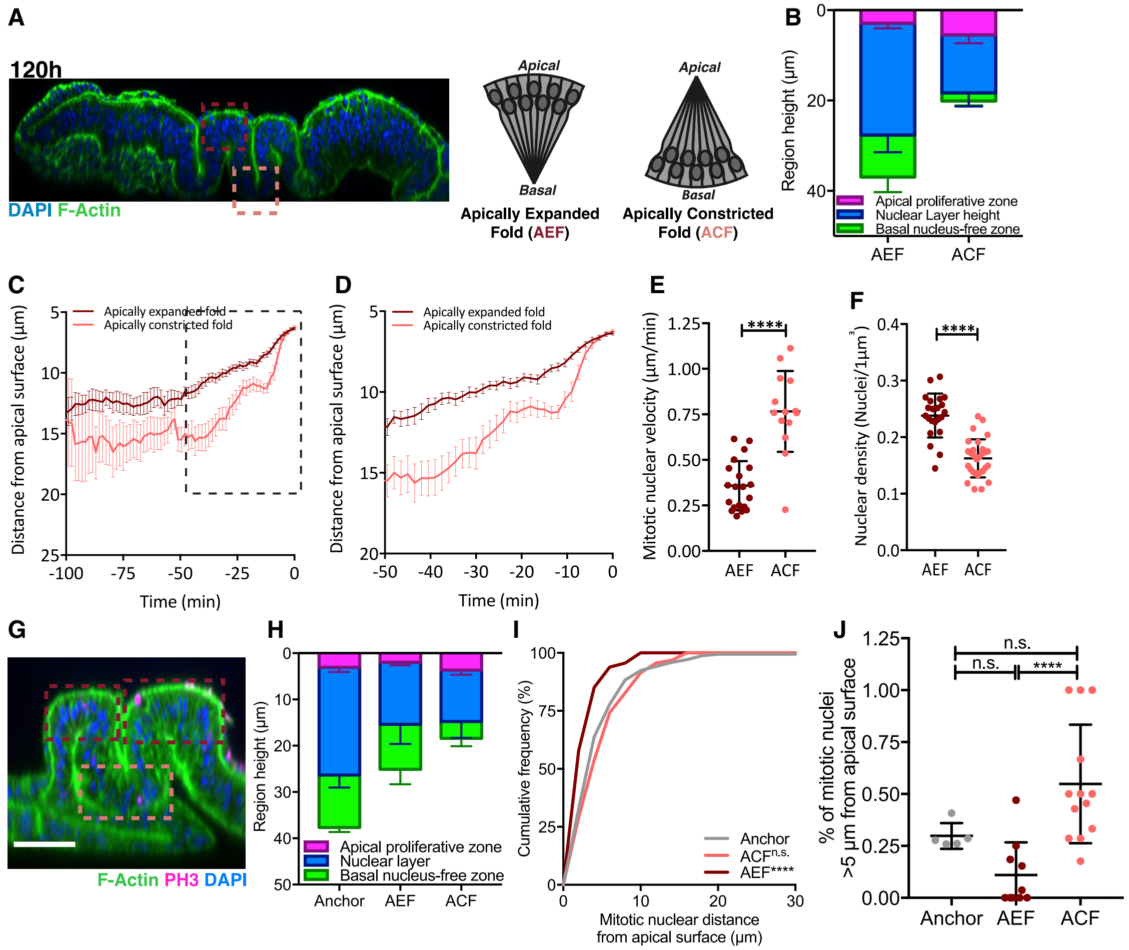

Figure 4. Tissue Folding Influences Mitotic Nuclear Positioning and Dynamics

(A) Cross-section of 120 h wing disc stained with DAPI (blue) and phalloidin (green), and schematic of epithelial topology in fold regions. Dark-red box, apically expanded fold; peach box, apically constricted fold.

(B) Epithelial apico-basal morphology for fold regions in wing discs expressing Sqh-GFP and His-RFP.

(C and D) Average mitotic nuclear track with directed apical movement for fold regions in wing discs expressing Sqh-GFP and His-RFP. Enlarged in inset (D). All tracks presented in Figures S4D and S4E.

(E) Mitotic nuclear velocity for the AEF and ACF for fold regions in wing discs expressing Sqh-GFP and His-RFP. The mean onset of upward movement was −14.8 and −11.1 min and the duration of upward movement was 10.8 and 6.9 min, respectively. n = 14 (AEF) and 20 (ACF) nuclear trajectories from 3 wing discs per condition.

(F) Nuclear density for fold regions in wing discs expressing Sqh-GFP and His-RFP. n = 3 wing discs.

(G) Representative image of mechanically buckled 120 h AEL wing disc. Folds marked as in (A). Scale bar, 10 μm.

(H) Epithelial apico-basal morphology for mechanically buckled regions.

(I) Cumulative frequency distribution of mitotic nuclear distance for mechanically buckled regions. Corresponding data in Figures S4G–S4I.

(J) Percentage of mitotic nuclei more than 5 μm from apical surface for mechanically buckled regions. n = 5 (anchored), 10 (AEF), and 13 (ACF).

Data for anchored discs also presented in Figures 3J–3N. Statistical significance: (E) and (F), unpaired t test (with Welch's correction in E); (I), Kolmogorov-Smirnov comparison of cumulative distribution; (J), one-way ANOVA. n.s., $p > 0.05$; ****$p < 0.0001$. Error bars: (C) and (D), mean ± SEM; (E), (F), (H), and (I), mean ± SD.

a milder reduction in mitotic nuclear velocity compared to *Rok-RNAi* cells located within clones (Figures 5M and 5N). We note, however, that *Rok-RNAi* cells within clones did not display the defects in apical nuclear positioning observed when Rok was depleted in the entire posterior domain (Figure 5M, *Rok-RNAi:Rok-RNAi* curve versus Figure 5C), which may be accounted for by the small clone sizes and the influence of wild-type neighboring regions. As *Rok-RNAi* reduces tissue tension [31], our findings suggest that the correct global mechanical state of the tissue may also be required for robust apical mitotic nuclear positioning.

Dependency on Dia for Apical Mitotic Nuclear Movement Increases with Development

Actin destabilizing drugs perturb apical mitotic positioning in other PSE [22–24]. Interestingly, formin-mediated actin polymerization is also essential for mitotic rounding when cells are under confinement [2–4, 8]. We therefore investigated the role of Dia, a

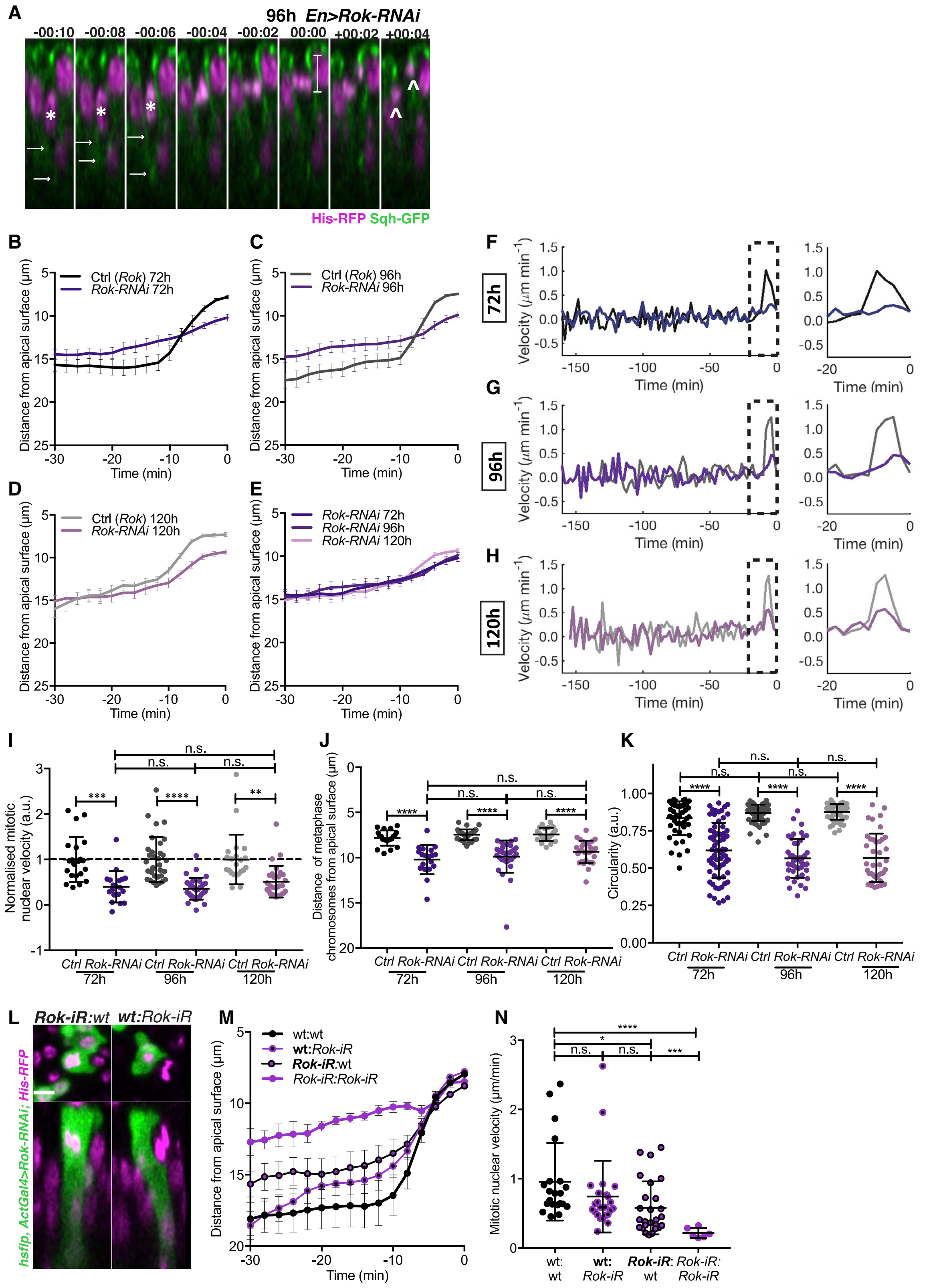

(legend on next page)

Drosophila formin, in regulating apical mitotic positioning, and whether this role changes as the tissue mechanical properties change. We first assessed the effects on mitotic positioning in fixed tissue (Figures S6A–S6D). Upon *dia-RNAi*, we found that compared to their age-matched controls, mitotic nuclei were distributed further from the apical surface at 96 and 120 h than at 72 h AEL (Figures S6E and S6F), indicating there may be development-specific requirements for Dia in apical mitotic positioning.

We then assessed live nuclear dynamics with reduced Dia expression. We observed a significant defect in mitotic nuclear movement upon *dia-RNAi* (Figures 6A–6E and S6G), and accordingly, late-phase instantaneous nuclear velocity and average mitotic nuclear velocity were reduced (Figures 6F–6H and S6J). Defects in nuclear dynamics also appeared to increase with development (Figures 6I and S6K).

To explore this further, we assessed nuclear positioning at metaphase. Metaphase nuclei were significantly further from the apical surface with *dia-RNAi*, and this defect increased through development (Figure 6J). Concurrently, we observed an incrementally worsening defect in mitotic rounding as development progressed (Figure 6K). Similarly, as with *Rok-RNAi*, when *dia-RNAi* nuclei failed to reach the apical surface, they exhibited defects in planar-oriented cell division (Figures 6A and S6G, white arrow heads).

To understand how Dia contributes to apically positioning mitotic nuclei, we generated small clones expressing *dia-RNAi*. We observed a consistent defect in mitotic nuclear dynamics in *dia-RNAi*-expressing cells, even in the presence of neighboring wild-type cells, while wild-type cells neighboring Dia-depleted cells were unperturbed (Figures 6L and 6M). Together, our results show that apical mitotic nuclear positioning increasingly depends on Dia in a cell-intrinsic manner, as development progresses.

Defects in Nuclear Positioning upon Dia Depletion Can Be Rescued by Mechanically Reducing Cell Density

Finally, we sought to assess whether the developmental increase in cell density generates the worsening defect in nuclear dynamics with *dia-RNAi*. We used our mechanical device to stretch and reduce cell density in *dia-RNAi*-expressing discs to test whether it could rescue defects in mitotic positioning (Figures 7A and S7A–S7C). We observed a greater shift in the distribution of PH3+ nuclei toward the apical surface upon stretching *dia-RNAi*-expressing discs than wild-type controls (Figures 7B–7E and S7D–S7F). Moreover, stretching reduced the proportion of translocating nuclei in *dia-RNAi* discs back to wild-type tissue levels (Figure 7E). Therefore, mechanically reducing cell density can rescue *dia-RNAi* induced defects, suggesting the greater requirement for Dia with development is due to increasing cell density.

DISCUSSION

Our findings demonstrate that nuclear movement required for apical mitosis in the pseudostratified *Drosophila* wing disc is mechanically regulated by tissue architecture. Using a combination of live-imaging and fixed tissue analysis, we show that nuclear movement during mitosis, rather than G2 [23, 26, 27], achieves apical mitotic positioning [24] (Figures 1, 2, S1, and S2). Our assessment of mitotic nuclear positions in fixed wing discs recapitulated differences in mitotic nuclear dynamics (Figures 1, S1, 2, and S2), allowing us to infer mitotic nuclear behaviors when genetic and mechanical perturbations were not amenable to live imaging (Figures 3 and 4G–4J).

We show that mitotic nuclear dynamics change as development progresses and tissue architecture changes (Figure 7F). Our data strongly suggest that it is the changes in nuclear density that strongly influence nuclear dynamics. However, we also observe a slower apical mitotic nuclear velocity in 72 h wing discs, compared to 96 h wing discs, even though pseudostratification and nuclear packing are less (Figures 1A–1E and 2A–2H). Cells of the 72 h wing disc are not clearly pseudostratified and display columnar-like morphology. It is therefore possible that mitotic rounding, which requires the lateral membranes to come

Figure 5. Rok Is Required for Apical Nuclear Movement at All Stages of Development

(A) Time-lapse images of mitotic nuclear movement for Sqh-GFP, His-RFP, and *EnGal4 > UAS-Rok-RNAi* wing discs at 96 h AEL. Arrows, cortical Sqh-GFP enrichment; asterisks, tracked nucleus; double-slash, skipped time-points. 00:00 min represents final metaphase. Bar: final metaphase distance. Arrowheads, apico-basal division.

(B–K) For 72, 96, and 120 h AEL, *EnGal4 > UAS-Rok-RNAi* wing discs. Shades of gray and black are control; shades of purple, *Rok*-RNAi. n = 19–33 nuclear trajectories acquired from 3 wing discs per stage (B–J), n = 40–67 cells from 3 wing discs (K).

(B–E) Average mitotic nuclear track (B, 72 h; C, 96 h; D, 120 h AEL; E, combined). Full time course shown in Figures S5J and S5K.

(F–H) Average instantaneous velocity measurements (F, 72 h; G, 96 h; H, 120 h AEL). Last 20 min shown in larger scale.

(I) Normalized mitotic nuclear velocity.

(J) Final metaphase distance from apical surface.

(K) Circularity at metaphase.

(L) Representative image of heat shock clones expressing *Rok-RNAi* (green). Left: *Rok-iR*:wt, *Rok-RNAi* mitotic cell bordering wild-type cells. Right: wt:*Rok-iR*, wild-type mitotic cell bordering *Rok-RNAi* cells.

(M) Average mitotic nuclear tracks for 96 h AEL wing discs expressing *Rok-RNAi* heat shock-induced clones.

(N) Mitotic nuclear velocity for 96 h AEL wing discs expressing *Rok-RNAi* heat shock-induced clones. The mean onset of apical movement was −9.0, −15.0, −11.1, and −20.75 min and the duration was 7.8, 14.1, 10.2, and 20.25 min for wt:wt, wt:*rok-iR*, *Rok-iR*:wt, and *Rok-iR:Rok-iR*, respectively. n = 21 (*wt:wt*), 24 (*wt:Rok-RNAi*), 26 (*Rok-RNAi:wt)*, and 6 (*Rok-RNAi: Rok-RNAi*) from 3 wing discs.

Other conditions in (L)–(N): wt:wt, wild-type cells surrounded by wild-type cells; *Rok-iR:Rok-iR*, *Rok-RNAi* cells surrounded by *Rok-RNAi* cells. Bold text: tracked nucleus.

Scale bars, 5 μm (A and L). Statistical significance: (I)–(K) and (N), one-way ANOVA. n.s., $p > 0.05$; *$p < 0.05$, **$p < 0.01$, ***$p < 0.001$, ****$p < 0.0001$. Error bars: (B)–(E) and (M), mean ± SEM; (I)–(K) and (N), mean ± SD.

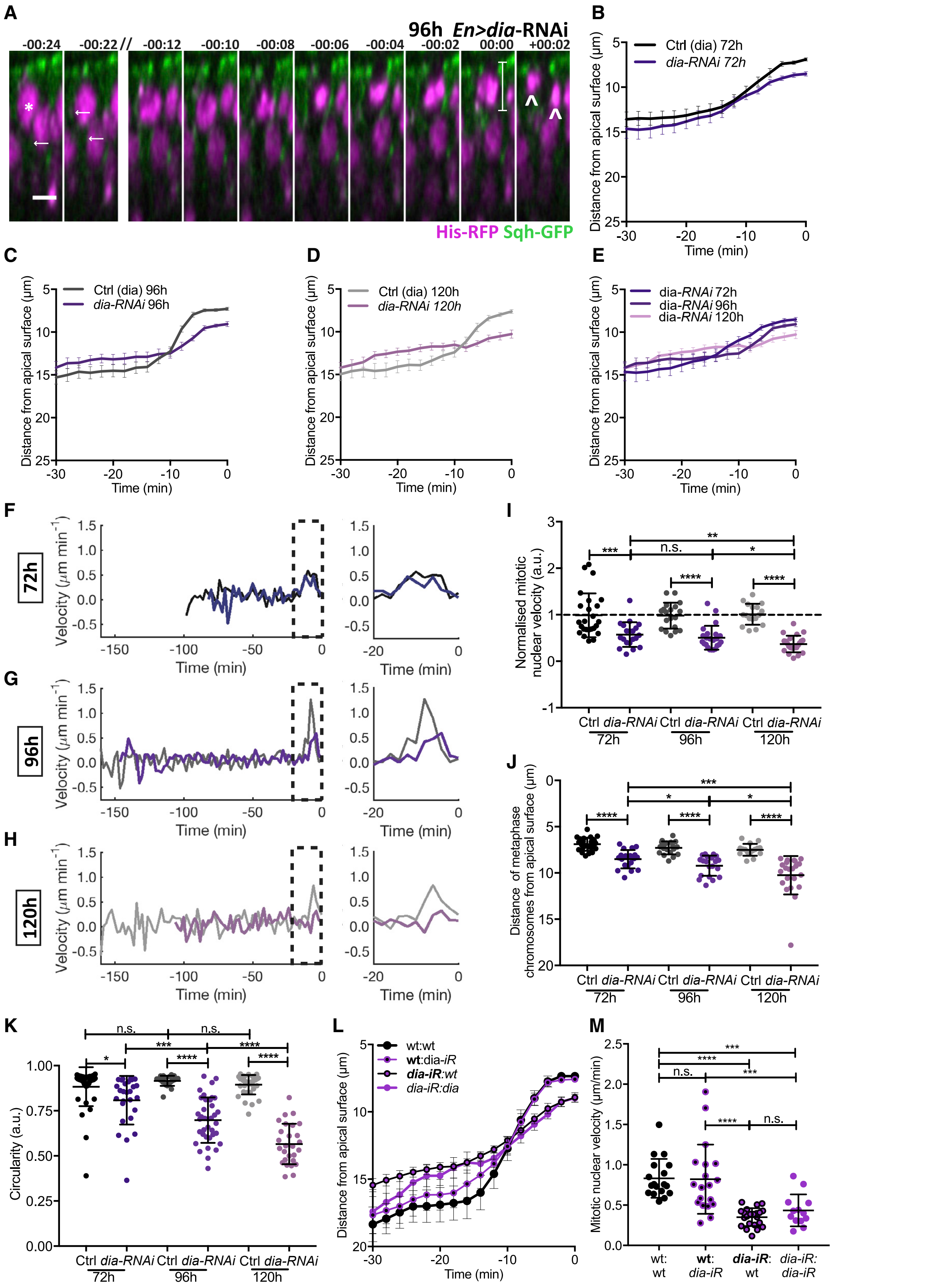

(legend on next page)

CellPress
OPEN ACCESS

together, is slower at 72 h than at 96 h AEL, when pseudostratification brings lateral membranes together. 72 h wing discs may therefore rely on additional mechanisms to promote efficient apical mitotic positioning.

We show that tissue folds can influence mitotic dynamics in a complex way, as they affect not only the curvature of the tissue, but also nuclear density (Figure 4). However, when we induce curvature without affecting nuclear density, we find positive apical curvature increases apical positioning compared to negative apical curvature (Figures 4G–4J). Tissue curvature and folding exist in many PSE [11, 28]. It has recently been shown that positive apical curvature of the zebrafish retina reduces apical migration compared to the flat hindbrain [28]. In light of our observations, it is possible that a mechanism by which nuclear density regulates nuclear movement overrides tissue curvature.

Finally, we show that reducing Rok and Dia in the wing disc reduces mitotic nuclear velocity and rounding, mispositions mitotic nuclei away from the apical surface, and results in sub-apical divisions (Figures 5 and 6). The essential requirement for Rok, a key activator of myosin II, does not differ between developmental stages and is therefore independent of tissue-packing density (Figure 7F). This supports a general requirement for myosin activity to support mitotic rounding [4, 32, 33], including in short and less densely packed epithelia [3, 8, 10]. In contrast, our data suggest there is a cell-intrinsic and increasing requirement for Dia as development progresses (Figure 7F), likely due to the increase in cell density (Figures 7A–7E). Dia is required for effective tension generation at the actomyosin cortex [5] and is particularly important for cell rounding in confinement [2–4]. Therefore, the increased dependency on Dia for apical nuclear positioning as cell density increases further supports our conclusion that nuclear movement and mitotic rounding are coupled (Figure 2). As cell density and confinement increase in PSE, greater force is required to generate mitotic rounding, hence the increasing need for Dia (Figure 7). Interestingly, it was also shown for the zebrafish retina, a curved PSE tissue, that Formin-like 3 protein has a specific role in nuclear migration by polymerizing an actin network under the nucleus that generates pushing forces, propelling the nucleus toward the apical side [28]. Therefore, formins may contribute to robust apical nuclear positioning via mechanistically distinct and complementary mechanisms.

In summary, our results reveal how the mechanical environment imposed on cells confined within a tissue can influence the molecular and cellular mechanisms that regulate nuclear movement to ensure robust apical mitosis. Further studies will be required to dissect exactly how feedback between tissue architecture and single-cell behaviors control global tissue growth and morphogenesis.

STAR★METHODS

Detailed methods are provided in the online version of this paper and include the following:

- KEY RESOURCES TABLE
- LEAD CONTACT AND MATERIALS AVAILABILITY
- EXPERIMENTAL MODEL AND SUBJECT DETAILS
  - Drosophila melanogaster
- METHOD DETAILS
  - Immunofluorescence
  - Live imaging
  - Wing disc stretch and compression
  - Heat shock clone induction
- QUANTIFICATION AND STATISTICAL ANALYSIS
  - Epithelial morphology
  - Nuclear density
  - Mitotic positioning in fixed tissue
  - Nuclear tracking
  - Lateral Myosin intensity
  - Determining mitotic nuclear velocity
  - Mean squared displacement profiles
  - Heat shock clone nuclear tracking
  - Statistical analysis
- DATA AND CODE AVAILABILITY

SUPPLEMENTAL INFORMATION

Supplemental Information can be found online at https://doi.org/10.1016/j.cub.2020.04.041.

Figure 6. Dependency on Dia for Apical Mitotic Nuclear Movement Increases with Development

(A) Time-lapse images of mitotic nuclear movement for Sqh-GFP, His-RFP, and *EnGal4 > UAS-dia-RNAi* wing discs at 96 h AEL. Marking as described in Figure 5A. Scale bar, 5 μm.

(B–K) For 72, 96, and 120 h AEL, *EnGal4 > UAS-dia-RNAi* wing discs. Shades of gray and black are control; shades of purple, *dia*-RNAi. n = 16–24 nuclear trajectories acquired from 3–4 wing discs per stage (B–J), n = 23–29 cells from 3 wing discs (K).

(B–E) Average mitotic nuclear track (B, 72 h; C, 96 h; D, 120 h AEL; E, combined). Full time course shown in Figures S6H and S6I.

(F–H) Average instantaneous velocity measurements (F, 72 h; G, 96 h; H, 120 h AEL). Last 20 min shown in larger scale.

(I) Normalized mitotic nuclear velocity.

(J) Final metaphase distance from apical surface.

(K) Circularity at metaphase.

(L) Average mitotic nuclear tracks for 96 h AEL wing discs expressing *dia-RNAi* heat shock-induced clones, labeled as in Figure 4L.

(M) Mitotic nuclear velocity for 96 h AEL wing discs expressing *dia-RNAi* heat shock-induced clones, labeled as in Figure 4L. The mean onset of apical movement was −11.2, −13.0, −19.8, and −16.7 min and the duration was 8.5, 10.1, 18.5, and 15.8 min for the wt:wt, wt:*dia-iR*, *dia-iR*:wt, and *dia-iR:dia-iR*, respectively. n = 18 (*wt:wt*), 19 (*wt:dia-RNAi*), 23 (*dia-RNAi:wt*), and 13 (*dia-RNAi:dia-RNAi*) from 3 wing discs.

Statistical significance: (I)–(K) and (M), one-way ANOVA. n.s., $p > 0.05$; *$p < 0.05$, ***$p < 0.001$, ****$p < 0.0001$. Error bars: (B)–(E) and (L), mean ± SEM; (I)–(K) and (M), mean ± SD.

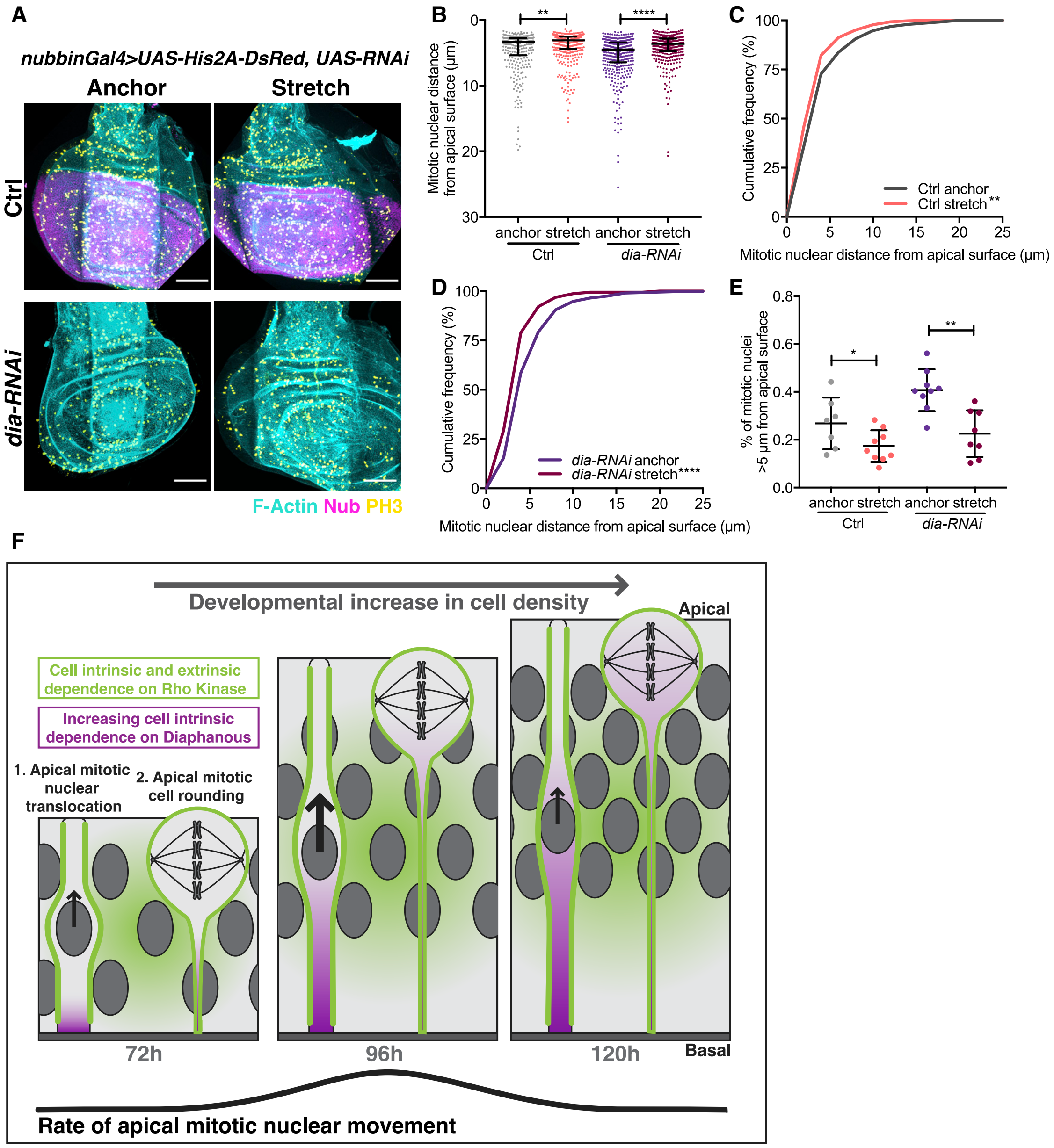

Figure 7. Defects in Nuclear Positioning upon Dia Depletion Can Be Rescued by Mechanically Reducing Cell Density

(A) Representative images of mechanically stretched 120 h wing discs, Top: *nubbin-Gal4, UAS-Ds-Red* control, Bottom: *nubbin-Gal4, UAS-Ds-Red, UAS-dia-RNAi*, stained with phalloidin (cyan) and anti-PH3 (yellow). Nubbin region shown for control, but present in *dia-RNAi*. Scale bar, 50 μm.

(B–D) Mitotic nuclear distance presented as a dot plot (B) and cumulative frequency distribution (C and D) for control and *dia-RNAi* wing discs in anchor and stretched conditions.

(E) Percentage of translocating mitotic nuclei more than 5 μm from the apical surface for control and *dia-RNAi* wing discs in anchor and stretched conditions. n = 7 (anchor) and 9 (stretch) wing discs for control and n = 9 (anchor) and 8 (stretch) wing discs for *dia-RNAi*. Corresponding morphology and normalized PH3 data in Figure S7.

(F) Schematic illustrating the influence of developmental increases in cell density and the role of Rok (green) and Dia (magenta) on apical mitotic nuclear movement.

Statistical significance: Kolmogorov-Smirnov comparison of cumulative distribution. *$p < 0.05$, **$p < 0.01$, ****$p < 0.0001$. Error bars: (B), median interquartile range; (E), mean ± SD.

ACKNOWLEDGMENTS

We thank R. Tetley for comments on the manuscript. We thank the Sanson, Baum, and Thompson labs for fly lines and the LMCB core microscopy facility for imaging support. N.J.K. is funded by an MRC PhD studentship. M.T. is funded by a Sir Henry Wellcome Fellowship (grant no. 103095). Y.M. is funded by an MRC Fellowship MR/L009056/1, a Lister Institute Research Prize, and EMBO Young Investigator Programme. E.K.P. is supported by the MRC

CellPress
OPEN ACCESS

Programme Award MC_UU_12018/5, an ERC starting grant 311637-MorphoCorDiv, and an ERC consolidator grant 820188-NanoMechShape. This work was also supported by MRC funding to the MRC LMCB University Unit at UCL, award code MC_U12266B.

AUTHOR CONTRIBUTIONS

N.J.K., E.K.P., and Y.M. conceptualized the project. N.J.K., A.C.Y., and N.H. performed experiments. N.J.K. and M.T. analyzed the data. N.J.K., M.T., E.K.P., and Y.M. wrote and edited the manuscript.

DECLARATION OF INTERESTS

The authors declare no competing interests.

Received: March 29, 2019
Revised: March 1, 2020
Accepted: April 20, 2020
Published: May 14, 2020

REFERENCES

1. Matthews, H.K., Delabre, U., Rohn, J.L., Guck, J., Kunda, P., and Baum, B. (2012). Changes in Ect2 localization couple actomyosin-dependent cell shape changes to mitotic progression. Dev. Cell *23*, 371–383.
2. Lancaster, O.M., Le Berre, M., Dimitracopoulos, A., Bonazzi, D., Zlotek-Zlotkiewicz, E., Picone, R., Duke, T., Piel, M., and Baum, B. (2013). Mitotic rounding alters cell geometry to ensure efficient bipolar spindle formation. Dev. Cell *25*, 270–283.
3. Rosa, A., Vlassaks, E., Pichaud, F., and Baum, B. (2015). Ect2/Pbl acts via Rho and polarity proteins to direct the assembly of an isotropic actomyosin cortex upon mitotic entry. Dev. Cell *32*, 604–616.
4. Ramanathan, S.P., Helenius, J., Stewart, M.P., Cattin, C.J., Hyman, A.A., and Muller, D.J. (2015). Cdk1-dependent mitotic enrichment of cortical myosin II promotes cell rounding against confinement. Nat. Cell Biol. *17*, 148–159.
5. Chugh, P., Clark, A.G., Smith, M.B., Cassani, D.A.D., Dierkes, K., Ragab, A., Roux, P.P., Charras, G., Salbreux, G., and Paluch, E.K. (2017). Actin cortex architecture regulates cell surface tension. Nat. Cell Biol. *19*, 689–697.
6. Stewart, M.P., Helenius, J., Toyoda, Y., Ramanathan, S.P., Muller, D.J., and Hyman, A.A. (2011). Hydrostatic pressure and the actomyosin cortex drive mitotic cell rounding. Nature *469*, 226–230.
7. Lancaster, O.M., and Baum, B. (2014). Shaping up to divide: coordinating actin and microtubule cytoskeletal remodelling during mitosis. Semin. Cell Dev. Biol. *34*, 109–115.
8. Sorce, B., Escobedo, C., Toyoda, Y., Stewart, M.P., Cattin, C.J., Newton, R., Banerjee, I., Stettler, A., Roska, B., Eaton, S., et al. (2015). Mitotic cells contract actomyosin cortex and generate pressure to round against or escape epithelial confinement. Nat. Commun. *6*, 8872.
9. Nakajima, Y., Meyer, E.J., Kroesen, A., McKinney, S.A., and Gibson, M.C. (2013). Epithelial junctions maintain tissue architecture by directing planar spindle orientation. Nature *500*, 359–362.
10. Chanet, S., Sharan, R., Khan, Z., and Martin, A.C. (2017). Myosin 2-induced mitotic rounding enables columnar epithelial cells to interpret cortical spindle positioning cues. Curr. Biol. *27*, 3350–3358.e3.
11. Grosse, A.S., Pressprich, M.F., Curley, L.B., Hamilton, K.L., Margolis, B., Hildebrand, J.D., and Gumucio, D.L. (2011). Cell dynamics in fetal intestinal epithelium: implications for intestinal growth and morphogenesis. Development *138*, 4423–4432.
12. Taverna, E., and Huttner, W.B. (2010). Neural progenitor nuclei IN motion. Neuron *67*, 906–914.
13. Strzyz, P.J., Matejcic, M., and Norden, C. (2016). Heterogeneity, cell biology and tissue mechanics of pseudostratified epithelia: coordination of cell divisions and growth in tightly packed tissues. Int. Rev. Cell Mol. Biol. *325*, 89–118.
14. Sauer, F.C. (1935). Mitosis in the neural tube. J. Comp. Neurol. *62*, 377–405.
15. Norden, C. (2017). Pseudostratified epithelia - cell biology, diversity and roles in organ formation at a glance. J. Cell Sci. *130*, 1859–1863.
16. Lee, H.O., and Norden, C. (2013). Mechanisms controlling arrangements and movements of nuclei in pseudostratified epithelia. Trends Cell Biol. *23*, 141–150.
17. Tsai, J.W., Lian, W.N., Kemal, S., Kriegstein, A.R., and Vallee, R.B. (2010). Kinesin 3 and cytoplasmic dynein mediate interkinetic nuclear migration in neural stem cells. Nat. Neurosci. *13*, 1463–1471.
18. Kosodo, Y., Suetsugu, T., Suda, M., Mimori-Kiyosue, Y., Toida, K., Baba, S.A., Kimura, A., and Matsuzaki, F. (2011). Regulation of interkinetic nuclear migration by cell cycle-coupled active and passive mechanisms in the developing brain. EMBO J. *30*, 1690–1704.
19. Spear, P.C., and Erickson, C.A. (2012). Apical movement during interkinetic nuclear migration is a two-step process. Dev. Biol. *370*, 33–41.
20. Hu, D.J., Baffet, A.D., Nayak, T., Akhmanova, A., Doye, V., and Vallee, R.B. (2013). Dynein recruitment to nuclear pores activates apical nuclear migration and mitotic entry in brain progenitor cells. Cell *154*, 1300–1313.
21. Baffet, A.D., Hu, D.J., and Vallee, R.B. (2015). Cdk1 activates pre-mitotic nuclear envelope dynein recruitment and apical nuclear migration in neural stem cells. Dev. Cell *33*, 703–716.
22. Norden, C., Young, S., Link, B.A., and Harris, W.A. (2009). Actomyosin is the main driver of interkinetic nuclear migration in the retina. Cell *138*, 1195–1208.
23. Leung, L., Klopper, A.V., Grill, S.W., Harris, W.A., and Norden, C. (2011). Apical migration of nuclei during G2 is a prerequisite for all nuclear motion in zebrafish neuroepithelia. Development *138*, 5003–5013.
24. Meyer, E.J., Ikmi, A., and Gibson, M.C. (2011). Interkinetic nuclear migration is a broadly conserved feature of cell division in pseudostratified epithelia. Curr. Biol. *21*, 485–491.
25. Rujano, M.A., Sanchez-Pulido, L., Pennetier, C., le Dez, G., and Basto, R. (2013). The microcephaly protein Asp regulates neuroepithelium morphogenesis by controlling the spatial distribution of myosin II. Nat. Cell Biol. *15*, 1294–1306.
26. Okamoto, M., Shinoda, T., Kawaue, T., Nagasaka, A., and Miyata, T. (2014). Ferret-mouse differences in interkinetic nuclear migration and cellular densification in the neocortical ventricular zone. Neurosci. Res. *86*, 88–95.
27. Nagasaka, A., Shinoda, T., Kawaue, T., Suzuki, M., Nagayama, K., Matsumoto, T., Ueno, N., Kawaguchi, A., and Miyata, T. (2016). Differences in the mechanical properties of the developing cerebral cortical proliferative zone between mice and ferrets at both the tissue and single-cell levels. Front. Cell Dev. Biol. *4*, 139.
28. Yanakieva, I., Erzberger, A., Matejčić, M., Modes, C.D., and Norden, C. (2019). Cell and tissue morphology determine actin-dependent nuclear migration mechanisms in neuroepithelia. J. Cell Biol. *218*, 3272–3289.
29. Pastor-Pareja, J.C., and Xu, T. (2011). Shaping cells and organs in Drosophila by opposing roles of fat body-secreted Collagen IV and perlecan. Dev. Cell *21*, 245–256.
30. Ma, M., Cao, X., Dai, J., and Pastor-Pareja, J.C. (2017). Basement membrane manipulation in Drosophila wing discs affects Dpp retention but not growth mechanoregulation. Dev. Cell *42*, 97–106.e4.
31. Duda, M., Kirkland, N.J., Khalilgharibi, N., Tozluoglu, M., Yuen, A.C., Carpi, N., Bove, A., Piel, M., Charras, G., Baum, B., and Mao, Y. (2019). Polarization of myosin II refines tissue material properties to buffer mechanical stress. Dev. Cell *48*, 245–260.e7.
32. Maddox, A.S., and Burridge, K. (2003). RhoA is required for cortical retraction and rigidity during mitotic cell rounding. J. Cell Biol. *160*, 255–265.
33. Kunda, P., Pelling, A.E., Liu, T., and Baum, B. (2008). Moesin controls cortical rigidity, cell rounding, and spindle morphogenesis during mitosis. Curr. Biol. *18*, 91–101.

34. Afshar, K., Stuart, B., and Wasserman, S.A. (2000). Functional analysis of the Drosophila diaphanous FH protein in early embryonic development. Development *127*, 1887–1897.

35. Schindelin, J., Arganda-Carreras, I., Frise, E., Kaynig, V., Longair, M., Pietzsch, T., Preibisch, S., Rueden, C., Saalfeld, S., Schmid, B., et al. (2012). Fiji: an open-source platform for biological-image analysis. Nat. Methods *9*, 676–682.

36. Bittig, T., Wartlick, O., González-Gaitán, M., and Jülicher, F. (2009). Quantification of growth asymmetries in developing epithelia. Eur Phys J E Soft Matter *30*, 93–99.

STAR★METHODS

KEY RESOURCES TABLE

REAGENT or RESOURCE	SOURCE	IDENTIFIER
Antibodies		
Donkey anti-mouse RRX	Jackson Immuno Research	115-295-003; RRID: AB_2338756
Goat anti-mouse Alexa 488	Life Technologies	A11029; RRID: AB_138404
Goat anti-rabbit Alexa 555	Abcam	ab150086; RRID: AB_2722519
Goat anti-rabbit Alexa 488	Life Technologies	A11034; RRID: AB_2576217
Mouse anti-Engrailed	Developmental Studies Hybridoma Bank	4D9; RRID: AB_528224
Rabbit anti-Diaphanous	[34]	N/A
Rabbit anti-pMRLC	Cell Signaling	3671S; RRID: AB_330248
Mouse anti-PH3	Millipore	3H10 05-806; RRID: AB_310016
Rabbit anti-PH3	Millipore	3H10 06-570; RRID: AB_1163440
Chemicals, Peptides, and Recombinant Proteins		
DAPI	Sigma-Aldrich	D8417
Fluoromount G slide mounting medium	SouthernBiotech	0100-01
Phalloidin Alexa-647	Life Technologies	A22287
Cell-Tak	Corning	354240
Shields and Sang M3 media	Sigma	S3652
Fetal Bovine Serum	Sigma	F4135
Penicillin-Streptomycin (10,000 U/mL)	Life Technologies	15140-122
20-Hydroxyecdysone	Sigma	H5142
Insulin solution from bovine pancreas, 10mg/mL	Sigma	I0516-5ML
16% w/v Formaldehyde	TAAB Laboratories	F017/3
GelPak, 6.5 mil	GelPak	PF-70-x4/6.5mil
Microposit EC solvent	Dow Chemicals	N/A
Su-8 2050	Microchem	Su-8 2050
SYLGARD 184 elastomer kit	Dow Corning	1673921
Triton X-100	Sigma	T8787
Bovine Serum Albumin (BSA)	Sigma	A7030
Experimental Models: Organisms/Strains		
yw;;;	BDSC	N/A
;;SqhGFP, HisRFP;	Y. Mao	N/A
;actGal4/CyO;;	B. Baum	N/A
;trol-RNAi;;	VDRC	GD/24549
UAS-trol;;;	Kyoto	DGRC 201233
;act > flp > Gal4, UAS-GFP; His-RFP;	Y. Mao	N/A
ywhsflp; Rok-RNAi;;	Y. Mao & VDRC	KK/104675
ywhsflp; dia-RNAi;;	Y. Mao & VDRC	KK/103914
;Bsg-GFP, His-RFP;;	Y. Mao & Kyoto	DGRC 115366
;EnGal4, ECad-GFP; UAS-NLS-Cherry;	Y. Mao & B. Sanson	N/A
;Rok-RNAi;;	VDRC	KK/104675
;dia-RNAi;;	VDRC	KK/103914
;enGal4; SqhGFP, HisRFP;	Y. Mao & B. Sanson	N/A
;nubGal4; UAS-His2A-DsRed;	B. Thompson	N/A

(*Continued on next page*)

Continued		
REAGENT or RESOURCE	SOURCE	IDENTIFIER
Software and Algorithms		
GraphPad Prism 7	GraphPad Software	N/A
Fiji	[35]	N/A
Microsoft Excel 2011	Microsoft	N/A
MATLAB 2016	MathWorks	N/A

LEAD CONTACT AND MATERIALS AVAILABILITY

Further information and requests for resources and reagents should be directed to and will be fulfilled by the Lead Contact, Yanlan Mao (y.mao@ucl.ac.uk). All unique/stable reagents and the custom code generated for the manuscript are available from the Lead Contact upon request.

EXPERIMENTAL MODEL AND SUBJECT DETAILS

Drosophila melanogaster

Fly stocks were raised in non-crowded conditions on standard cornmeal molasses fly food medium at 25°C. Fly food consisted of, per 1L, 10 g agar, 15 g sucrose, 33 g glucose, 35 g years, 15 g maize meal, 10 g wheat germ, 30 g treacle, 7.22 g soya flour, 1g nipagin, 5ml propionic acid.

Male and female larvae were dissected at early to late 3rd instar development (approximately 72-120 h AEL) for experiments. For developmental staging, flies were staged every 24 h. Appropriate larva were selected for dissection and wing disc morphology used to refine staging. 72 h discs were defined by flat, tear drop shaped epithelia; 96 h wing discs had all three folds formed but their surface area measures substantially smaller than the later stages (30,000μm^2); 120 h wing discs have all three folds, are enlarged (46,000μm^2) and exhibit a condensation of cells at the dorsal-ventral midline. For *trol-RNAi* mutants, in which disc morphology differs, discs were staged to the timing of the wild-type 96 h counterparts.

METHOD DETAILS

Immunofluorescence

Larva and forceps were washed in 70% ethanol and PBS. Larva were transferred to dissecting medium (Shields and Sang M3 media, 2% FBS, 1% pen/strep, 3 ng/mL hydroxyecdysone and 0.05 units/l insulin) and their wing discs extracted for up to 15 min. Discs were fixed for 30 min in 4% formaldehyde-PBS at room temperature. Fixed tissue was washed 4x10 min PBT (PBS, 0.3% Triton X-100) and 4x10 min PBT-BSA (PBT, 0.5% BSA). Primary antibody at appropriate concentrations was incubated overnight. Washed were repeated and secondary antibody, with DAPI and Phalloidin incubated for 1-2 h at room temperature. Discs were washed for 3x20 min PBT and 3x quick rinse with PBS. Discs were mounted in Fluoromount G slide mounting medium (Southern Biotech) for imaging.

Antibodies and Dyes

Mouse anti-PH3 (Millipore 3H10 05-806), 1:500. Rabbit anti-PH3 (Millipore 3H10 06-570), 1:150. Mouse anti-Engrailed (DSHB, 4D9), 1:50. Rabbit anti-phospo-Myosin (3671, Cell Signaling), Rabbit anti-Diaphanous (Afshar et al., 2000), Goat anti-mouse Alexa 488 (Life Technologies, A11029), 1:500. Donkey anti-mouse-RRX (JacksonImmunoResearch), 1:500. Goat anti-rabbit Alexa 488 (Life Technologies, A11034), 1:500. Goat anti-rabbit Alexa-555 (Abcam, ab150086), 1:500. DAPI (Sigma-Aldrich, D8417), 1:1000. Alexa Fluor 647 Phalloidin (Cell Signaling, 8953S and A22287, Life Technologies) (1:20).

Imaging

Samples were imaged using a Leica SP5 or SP8 inverted confocal microscope with a 40X objective, 1-2X zoom, 0.35 μm depth resolution and 1024^2 or 512^2 XY pixel resolution.

Live imaging

Pouch region sample preparation

Discs were dissected as described above. Discs were transferred in dissecting media to Fluoro glass-bottomed dish and positioned apical side down onto a 0.4 μl line of Cell-Tak (Corning, 354240) (previously dried onto glass-bottom on heat plate set to 29°C). A further 1 mL of dissecting media was added before sealing the dish with parafilm.

Fold region sample preparation

Discs mounted as described for pouch region with the exception of the basal fold, which is instead mounted basal side down.

Imaging

All live imaging was carried out using an inverted Zeiss LSM880 microscope with 40X oil lens, 2X zoom, 512by256 XY resolution and 0.5 μm depth resolution for confocal stack imaging. The LSM880 Airyscan detector was used in confocal setting for sensitive

imaging. Laser settings and resolution were identical for each individual experimental group and for *enGal4*,*RNAi* expression experiments, both tissue regions were in the same field of view.

Wing disc stretch and compression

Larva were dissected as for immunofluorescence and live imaging experiments. The assembly and use of the stretcher device has been previously described [31]. Specific to this work, discs were loaded into the device so the manipulation was applied bi-directionally along the anterior-posterior axis and held for 30 min. Compression was applied by pre-stretching PDMS membranes prior to disc loading. The loaded PDMS was then relaxed to compress the tissue along the anterior-posterior axis. This manipulation also resulted in the occasional buckled tissue, which were post-sorted for analysis. Tissues were fixed directly within the device with 4% PFA in PBS, for 15 min before transferring discs to a glass-well dish for a further 15 min of fixing. Immunostaining is carried out as described above.

Heat shock clone induction

For *Rok-RNAi* and *dia-RNAi* flipout clones, *yw hs.flp / +; actin-FRT-stop-FRT- Gal4, UAS-GFP / Rok-RNAi; His-RFP / + and*, *yw hs.flp / +; actin-FRT-stop-FRT- Gal4, UAS-GFP / dia-RNAi; His-RFP /* + larvae were heat shocked at 37°C for 15 min at 72 h AEL and dissected for imaging 24 h later (96 h AEL).

QUANTIFICATION AND STATISTICAL ANALYSIS

Epithelial morphology

To measure the height of the epithelia, nuclear layer, nuclear region, apical proliferative zone and the basal nucleus free zone, confocal stack images were "resliced" in ImageJ along the dorsal-ventral axis. For yw, mechanically-perturbed and *trol-RNAi* discs, measurements were taken from the anterior third, center and posterior third of the pouch region. Three intensity profile measurements from the apico-basal axis were obtained from the DAPI channel as shown in Figure S1A, left panel. For mutant perturbation with *EnGal4, ECad-GFP; UAS-NLS-Cherry* three measurements from two cross-sections in the anterior and posterior compartment were acquired, representing the control and mutant respectively. Each respective measurement was obtained by the measurements for the epithelial height, the starting and the final position of the nuclear layer as illustrated in Figure S1A, right panel. The number of nuclei within the nuclear layers was measured by the counting the number of peaks for each intensity profile. The average value of the regions was calculated for each disc and subject to an independent t test and presented as a plot. The values for the apical proliferative zone, the nuclear layer and the basal nucleus free zone were presented as "Stacked Bars" in Prism to reflect the epithelium morphology for the disc condition (For example see Figures 1B and 1B′).

Nuclear density

Density was calculated as in Bittig et al., 2009 [36]. 3-5 segments of 50-90mm^2 apical surface areas were sampled per disc and "resliced" to present the z sectioning in ImageJ. The volume of the nuclear region was calculated by multiplying the apical surface area measurement by the height of the nuclear layers. 3 wing discs were measured per condition, and each density measurement collated and presented to demonstrate the range across the wing disc.

Mitotic positioning in fixed tissue

In the pouch region of the wing disc, the mitotic distance was obtained by measuring the distance from the center of the PH3+ nuclei to the apical surface, as marked by F-Actin as illustrated in Figure 1E. To obtain a position with respect to the region in which the mitotic nuclei could translocate, the mitotic distance was divided by the average height of the nuclear region as illustrated in Figure S1F. To plot the distribution of nuclei for each measurement type, the data points from each age-matched disc of an experimental group were pooled. The distribution of the mitotic positions were statistically compared using the non-parametric Kolmogorov-Smirnov comparison of cumulative distribution. For scatterplots, the y axis scale was reversed so the PH3 position would represent the apico- basal organization of the wing disc cells as depicted in the images of the epithelium.

Nuclear tracking

Confocal stack, time-lapse images were corrected for drift using the 3D drift correction plugin in ImageJ. The corrected imaging was the "resliced" to present the apico-basal axis. Nuclei were manually tracked by measuring the distance from the basal side of the nucleus to the apical surface at each time-step, with nuclei marked by His-RFP and the apical surface marked by Sqh-GFP expression. Mitotic cells were identified by tracking backward from when the cell is in a final metaphase state, prior to anaphase chromosome separation. Excel and Prism were used to revers and align tracks and present trajectories. Tracks were statistically analyzed as described below.

For montage images, the drift corrected time-lapse imaging were subject to background subtraction (rolling ball radius 30, ImageJ) and 3D Gaussian filtering (1.0 pixel diameter) before presenting.

Lateral Myosin intensity

The lateral membrane of the 72 h wing disc cells were traced in FIJI with a 2 pixel width and the mean intensity of Sqh-GFP measured. When the lateral membrane Sqh-GFP is indistinguishable from the background cytoplasmic signal, as occurs prior to mitotic onset, two 5μm line measurements were taken from either side of the nucleus. The intensity measurements were then normalized by dividing by the average intensity of the time points collected before –30:00, where 0:00 is metaphase, and associated with stochastic nuclear movement. The corresponding nuclei were also tracked for presenting with the myosin intensity analysis.

Determining mitotic nuclear velocity

The start of apical mitotic movement was determined by the initiation of persistent apical movement and cortical Sqh-GFP enrichment, while the final position was determined at the metaphase, assessed from the nuclear tracks and the respective live imaging. The distance between these two points was divided by the length of time taken to calculate the mitotic nuclear velocity for each track.

To compare the fold effect of *dia* and *Rok* RNAi expression on mitotic nuclear velocity, each measurement was divided by the mean mitotic nuclear velocity measurement for the control condition at the respective disc age (the average of all tracks, across all discs at the respective developmental stage).

Mean squared displacement profiles

The mean squared distance of a trajectory for a given time lag Δt was calculated from the average of squared displacement between all data couples separated by Δt. For a trajectory of N data points p and a data collection interval of δt, the MSD for lag $\Delta t = n\,\delta t$ becomes:

$$MSD(\Delta\, t) = \frac{1}{N-n}\sum_{i=1}^{N-n}(p((n+i)\delta t) - p(i\,\delta t))^2$$

The MSD for increasing time lag were calculated for each individual nucleus sub-trajectory toward the apical surface. The sub-trajectories are defined as the early and late phase nuclear migration. The transition from the early to the late phase was determined manually as the onset of persistent apical movement and enrichment of cortical Sqh-GFP. The late phase was truncated at metaphase, when no further apical movement occurred. The maximum lag is limited by the length of the late phase trajectory in each developmental time and mutant dataset.

Then, with the log-log linearized form of a power law model, $\ln(MSD(\Delta t)) = \ln(2D) + a\ln(\Delta t)$, the motion of the nuclei is characterized. In this model, D is the diffusion constant of the observed particles. Here, a slope below 1.0 would indicate constrained diffusion, equal to 1.0 would indicate stochastic random diffusion and any value above 1.0 would indicate super (directional) diffusion, with reaching 2.0 indicating ballistic motion.

For clarity, the data is displayed in MSD/2D versus time lag form, where the logarithm of the diffusion constant parameter is obtained from the intercept of fitted line. Linear regression is used to fit lines to the log-log plots of MSD versus time lag for the early and late phases in each of the wing disc age groups, as identified above. The change in the slope between early and late phase for each age is utilized as an indicator of how the diffusive behavior changes between the early and late phases of mitotic nuclei motion.

Heat shock clone nuclear tracking

For heat-shock flipout clones of *Rok-RNAi* and *dia-RNAi,* compartment conditions were determined as; wt-wt, tracked wildtype mitotic cells shared 100% of their apical perimeter with other wildtype cells at the apical surface; wt-RNAi, tracked wildtype cells had 50%–100% of their apical perimeter adjoined to a GFP+, RNAi expressing cell; RNAi-wt, tracked GFP+, RNAi expressing cell shared 50%–100% of their apical cell perimeter with wildtype cells and RNAi-RNAi, GFP+, RNAi expressing cell shared 100% of their apical cell perimeter with other GFP+, RNAi expressing cells.

Statistical analysis

Microsoft Excel 2011, MATLAB 2016 and Prism 7 Software were used to present data and conduct statistical analysis. The respective statistical tests, measures of center and dispersion, and n numbers are described in the figure legends.

For epithelial morphology analysis, a minimum of three discs were used per condition. For fixed tissue analysis of mitotic position, a minimum of 5 discs were used per condition. For live nuclear tracking, a minimum of three discs were used per condition. The following statistical significance cut off was applied: n.s. $p > 0.05$, * $p < 0.05$, **$p < 0.01$, ***$p < 0.01$, ****$p < 0.0001$. No tests were conducted to measure statistical power or normality of distributions.

DATA AND CODE AVAILABILITY

The authors declare that the data supporting the findings of this study and custom code generated for the manuscript are available from the Lead Contact upon request.

Current Biology, Volume *30*

Supplemental Information

Tissue Mechanics Regulate Mitotic Nuclear

Dynamics during Epithelial Development

Natalie J. Kirkland, Alice C. Yuen, Melda Tozluoglu, Nancy Hui, Ewa K. Paluch, and Yanlan Mao

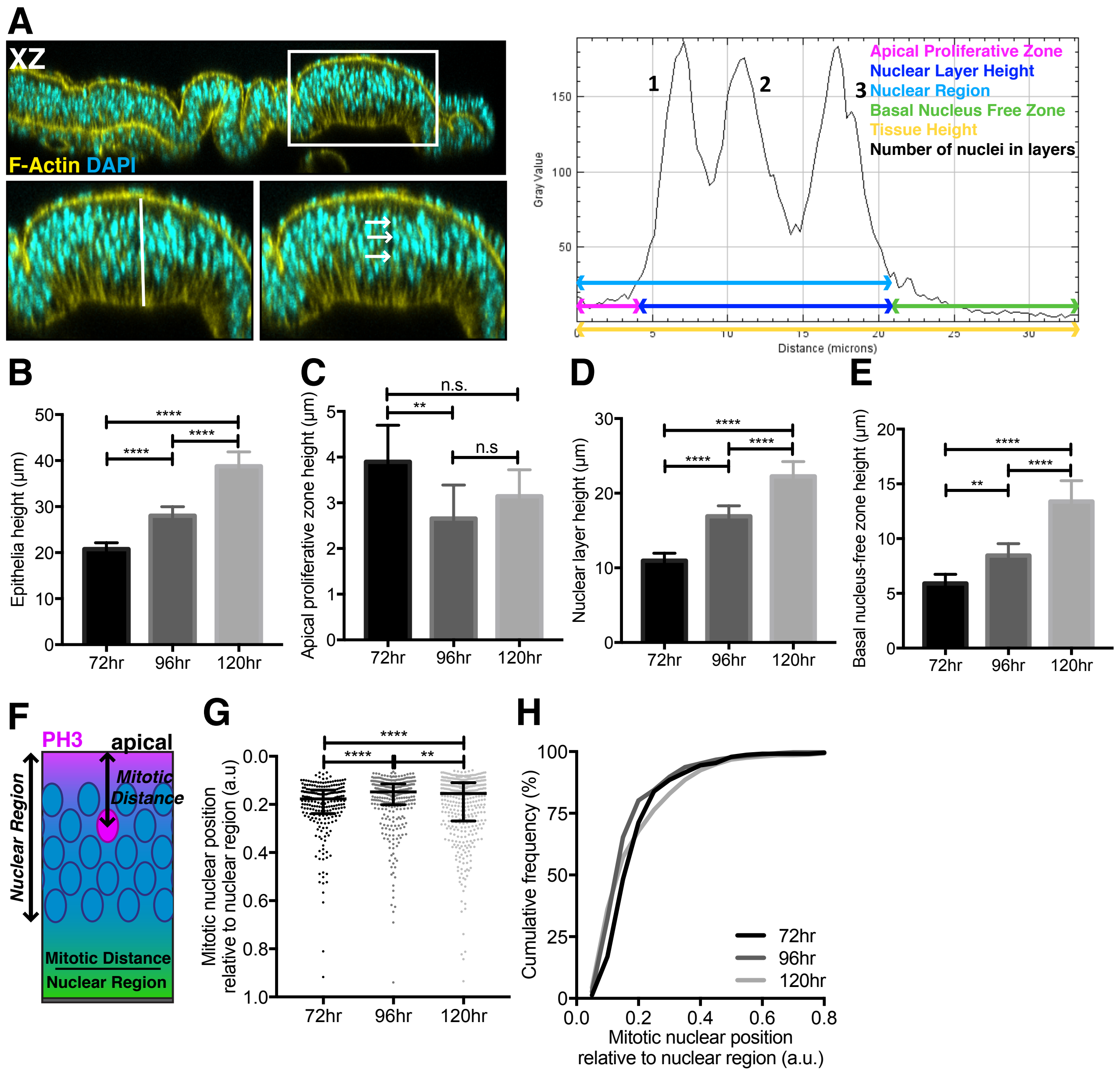

Figure S1. Changes in the wing disc architecture during development are associated with distinct patterns of mitotic nuclear positioning. Related to Figure 1.

(A) Left panel, lateral cross section of wing disc fixed and stained with DAPI (cyan) and phalloidin (yellow). White box highlights inset pouch region shown below. White line in lower left inset highlights the apico-basal cross-section used to generate the intensity plot displayed in the right panel. Lower right inset: white arrows mark the nuclei shown as peaks in the right panel, used for counting nuclei number in nuclear layer. Right panel: labelled intensity plot illustrating quantification for Figure 1B-C and S1B-E. (B-E) Epithelial and sub-region height, also presented in Figure 1C. (F) Schematic showing mitotic nuclear position relative to the nuclear region as in S1F-H. (G-H) Normalised PH3+ distance from Figure 1G-H presented as dot plot (G) and cumulative frequency distribution (H). Average nuclear region height calculated per disc. (B-E, G-H) n = 8 (72hr), 8 (96hr) and 5 (120hr) wing. Statistical significance: B-E, one-way ANOVA; G, Kolmogorov-Smirnov comparison of cumulative distribution. n.s.>0.05, **p<0.01, ****p<0.0001. Error bars: B-E, mean ± SD; G, median ± interquartile range.

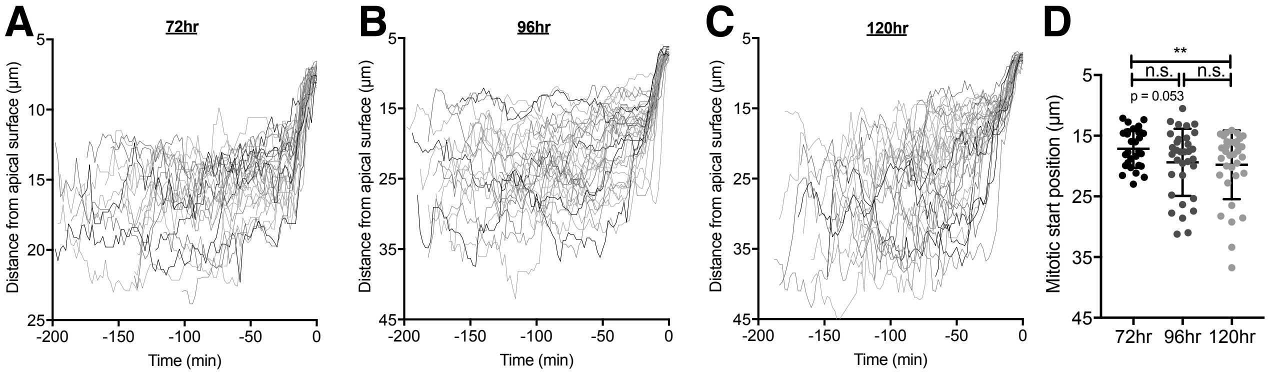

Figure S2. Mitotic nuclear dynamics depend on tissue architecture. Related to Figure 2.
(A-C) Individual nuclei tracks from wing disc pouch region at 72, 96 and 120 hours AEL, expressing Sqh-GFP and His-RFP, obtained by measuring distances illustrated in Figure 2A-C. 0 minutes is defined by metaphase nucleus prior to anaphase separation. (D) The position at the onset of apical nuclear movement, used to calculate mitotic nuclear velocity in Figure 2D. Statistical significance: one-way ANOVA. Error bars: mean ± SD. (A-D) n = 28, 33 and 34 nuclear trajectories from 3 (72hr), 3 (96hr) and 4 (120hr) wing discs respectively. n.s. $p>0.05$, **$p<0.01$.

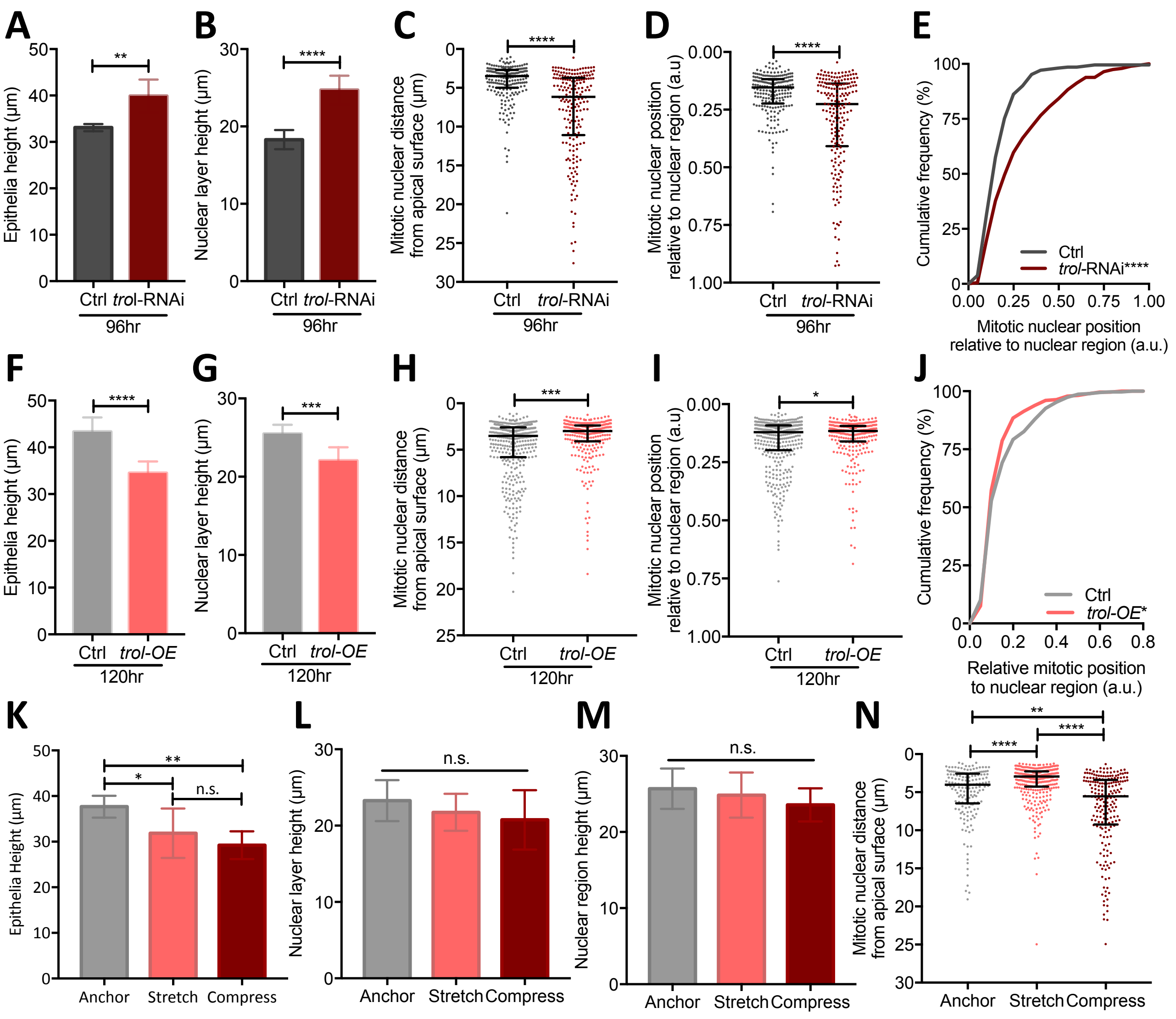

Figure S3. Perturbing cell density influences mitotic nuclear positioning. Related to Figure 3.

(A, B) Quantification of apico-basal epithelial morphology for control (*actin*Gal4) and *UAS-trol-RNAi* (*trol-RNAi*) wing discs, corresponding to Figure 3B. (C) Scatter plot of mitotic nuclear distance from the apical surface for control and *trol-RNAi* wing discs, corresponding to Figure 3D. (D, E) Mitotic nuclear position relative to the nuclear region for control and *trol-RNAi* expressing wing discs presented as scatter plot (D) and cumulative frequency distribution (E). (F, G) Quantification of apico-basal epithelial morphology for control and *UAS-trol (trol-OE)* wing discs, corresponding to Figure 3F. (H) Scatter plot of mitotic nuclear distance from the apical surface for control and *trol-OE* wing discs, corresponding to Figure 3H. (I, J) Mitotic nuclear position relative to the nuclear region for control and *trol-OE* expressing wing discs presented as scatter plot (I) and cumulative frequency distribution (J). (K-M) Quantification of apico-basal epithelial morphology in the wing disc pouch region upon mechanical perturbation as illustrated in Figure 3E. (N) Scatter plot of mitotic nuclear distance from the apical surface upon mechanical perturbation, corresponding to Figure 3I. (A-E) n = 6 (control) and 7 (*trol-RNAi*) wing discs. (F-J) n = 8 (control) and 11 (*trol-OE*) wing disc discs. (K-N) n = 3 (anchored), 8 (stretched) and 6 (compressed) wing discs. Statistical significance: A, B, F, G, unpaired t-test; C-E, H-J, N, Kolmogorov-Smirnov comparison of cumulative distribution; K-M, one-way ANOVA. *p<0.05, **p<0.01, ***p<0.001, ****p<0.0001. Error bars: A, B, F, G, K-M, mean ± SD; C, D, H, I, N, median ± interquartile range.

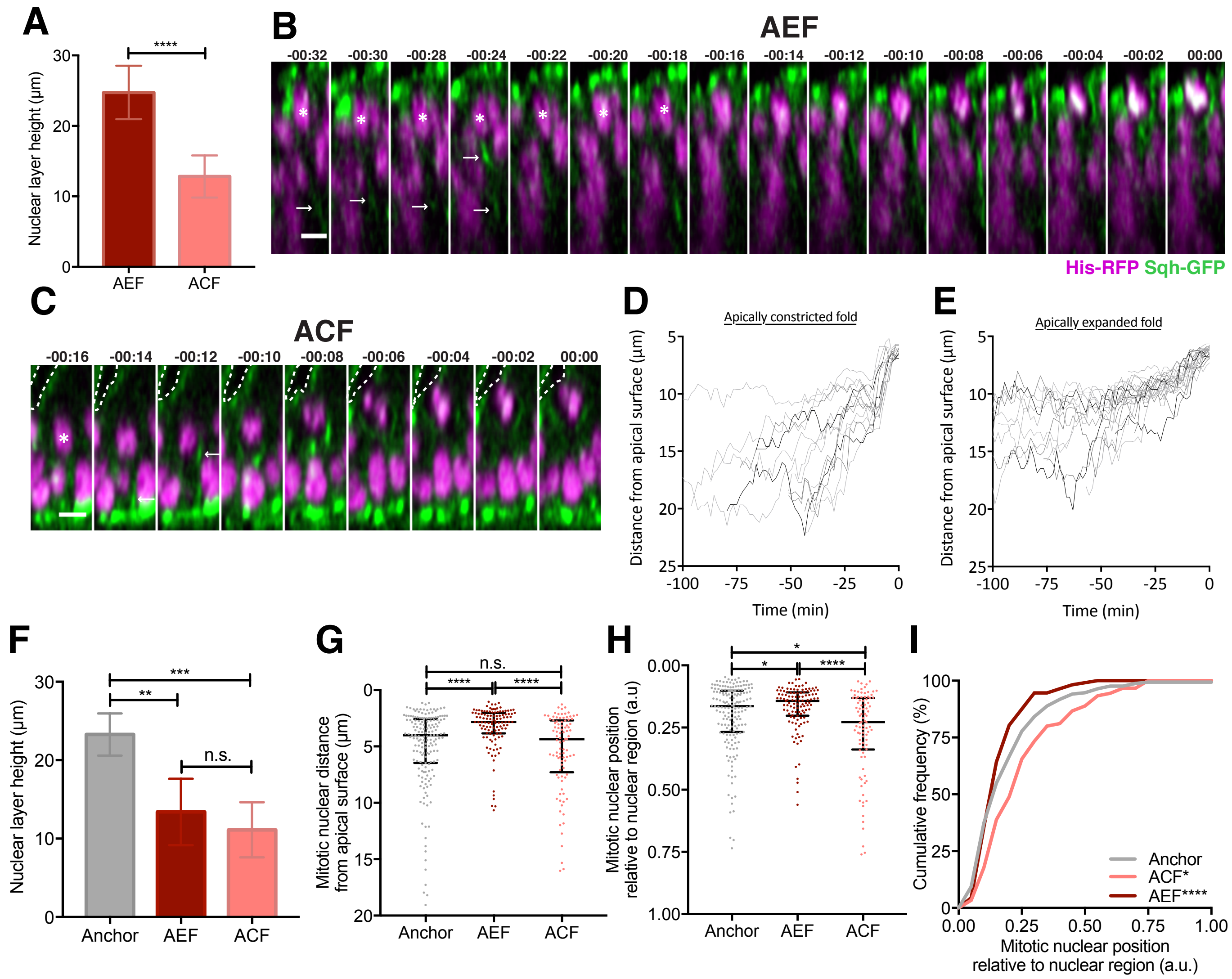

Figure S4. Tissue folding influences mitotic nuclear positioning and dynamics. Related to Figure 4.

(A) Nuclear layer height measurements from Figure 4B. (B, C) Representative time-lapses of mitotic nuclei moving apically in the distinct fold regions, the apically expanded fold (AEF) and apically constricted fold (ACF), in wing discs expressing Sqh-GFP and His-RFP. White arrows indicate cortical myosin enrichment. White asterisks mark the tracked nucleus when in crowded regions. White dashed line in C represents apical lumen region. (D, E) Individual nuclei tracks from the wing disc fold regions averaged in Figure 4C, D. (F) Nuclear layer height measurements from Figure 4H. (G) Mitotic nuclear distance from apical surface presented as dot plot, corresponding to Figure 4I. (H, I) Distance of mitotic nuclei from apical surface relative to nuclear region presented as dot plot (H) and cumulative frequency distribution (I). (A, D, E) n = 14 (AEF) and 20 (ACF) nuclear trajectories from 3 wing discs per condition. (F-I) n = 5 (anchored), 10 (AEF), 13 (ACF) wing discs. Scale bars: A,B, 5 μm. Statistical significance: A, unpaired t-test; F, one-way ANOVA; G-I, Kolmogorov-Smirnov comparison of cumulative distribution. *$p<0.05$, **$p<0.01$, ***$p<0.001$, ****$p<0.0001$. Error bars: A,D, mean ± SD; G-I, median ± interquartile range.

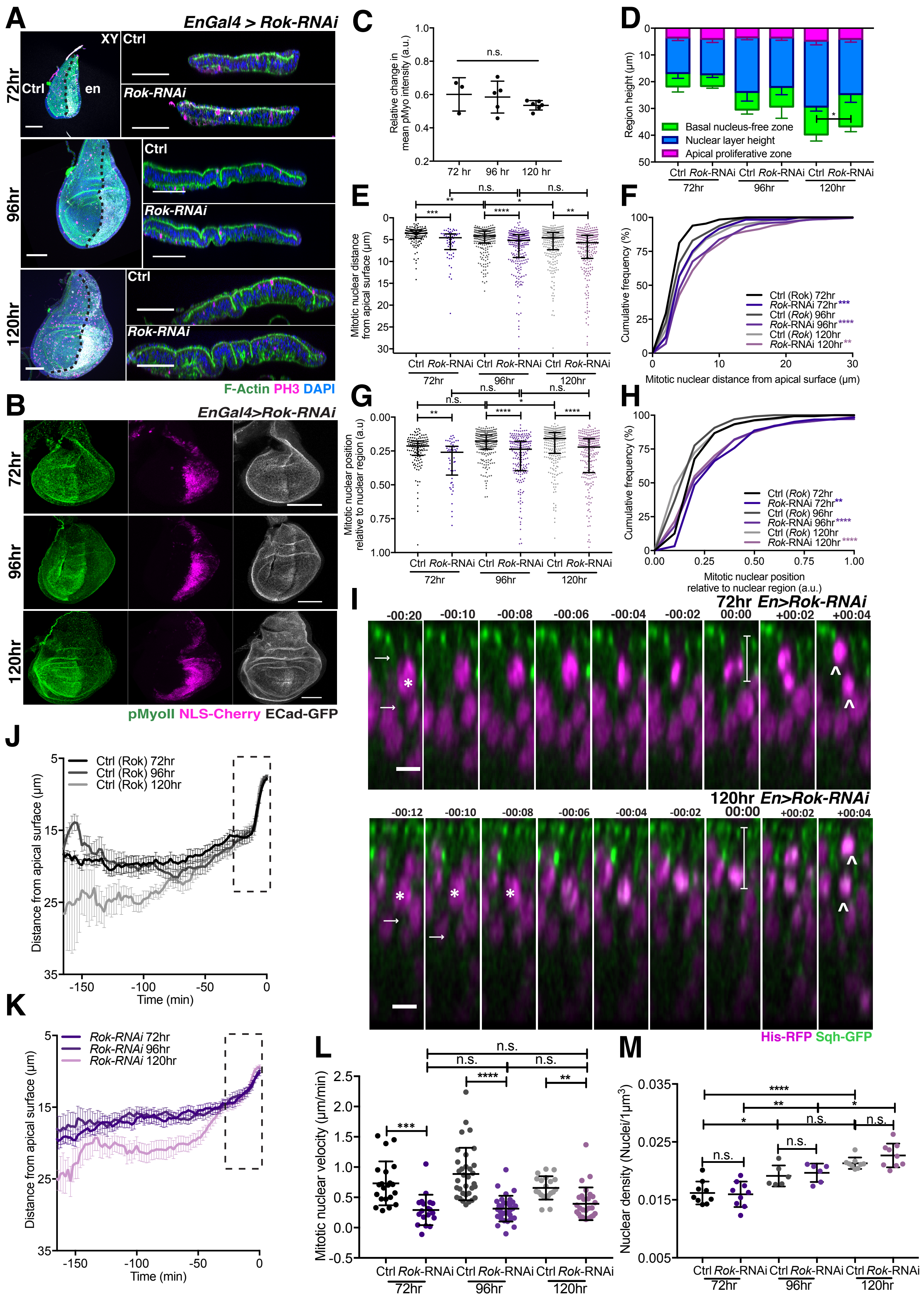

A
EnGal4 > Rok-RNAi
72hr
96hr
120hr
XY
Ctrl
en
Rok-RNAi
F-Actin PH3 DAPI
B
EnGal4>Rok-RNAi
pMyoII NLS-Cherry ECad-GFP
C
Relative change in mean pMyo intensity (a.u.)
n.s.
72 hr
96 hr
120 hr
D
Region height (μm)
Basal nucleus-free zone
Nuclear layer height
Apical proliferative zone
Ctrl Rok-RNAi Ctrl Rok-RNAi Ctrl Rok-RNAi
72hr
96hr
120hr
E
Mitotic nuclear distance from apical surface (μm)
F
Cumulative frequency (%)
Ctrl (Rok) 72hr
Rok-RNAi 72hr***
Ctrl (Rok) 96hr
Rok-RNAi 96hr****
Ctrl (Rok) 120hr
Rok-RNAi 120hr**
Mitotic nuclear distance from apical surface (μm)
G
Mitotic nuclear position relative to nuclear region (a.u)
H
Ctrl (Rok) 72hr
Rok-RNAi 72hr**
Ctrl (Rok) 96hr
Rok-RNAi 96hr****
Ctrl (Rok) 120hr
Rok-RNAi 120hr****
Mitotic nuclear position relative to nuclear region (a.u.)
I
72hr En>Rok-RNAi
120hr En>Rok-RNAi
His-RFP Sqh-GFP
J
Ctrl (Rok) 72hr
Ctrl (Rok) 96hr
Ctrl (Rok) 120hr
Distance from apical surface (μm)
Time (min)
K
Rok-RNAi 72hr
Rok-RNAi 96hr
Rok-RNAi 120hr
Distance from apical surface (μm)
Time (min)
L
Mitotic nuclear velocity (μm/min)
M
Nuclear density (Nuclei/1μm3)

Figure S5. Rok is required for apical nuclear movement at all stages of development. Related to Figure 5.

(A) Representative images of wing disc morphology with *Rok-RNAi* expression in *engrailed* domain (posterior, *en*) at different developmental stages. *Rok-RNAi* expression marked by UAS-NLS-Cherry in the posterior region (grey), phalloidin (green), anti-PH3 (magenta) and DAPI (blue). Black dashed line shows anterior-posterior boundary. (B) Representative images of phospho-Myosin-II (pMyo) staining in *EnGal4>Rok-RNAi* wing discs. Green staining shows anti-pMyoII, anterior region with UAS-NLS-Cherry in magenta and E-Cadherin-GFP in grey. (C) Relative change in pMyo staining mean intensity in *Rok-RNAi* region compared to control region. (D) Epithelial morphology measurements for wing discs in (E-H). Statistical difference between control and *Rok-RNAi* for only nuclear layer height at 120hr AEL. (E, F) Mitotic nuclear distance from apical surface for control and *Rok-RNAi* wing discs presented as dot plot (E) and cumulative frequency distribution (F). (G, H) Data in (E, F) normalised to nuclear region. (I) Representative images of *Rok-RNAi* mitotic nuclei moving apically within posterior pouch region at 72hr and 120hr AEL. Wing discs express Sqh-GFP, His-RFP and *EnGal4>UAS-Rok-RNAi*. Arrows: cortical myosin enrichment regions. Asterisks: tracked nuclei. Arrow heads: apico-basal cell division. Line bar: distance of metaphase nucleus from apical surface at 00:00 mins, with values presented in Figure 5I. (J, K) Full time-course for average nuclear tracks for anterior control (J) and posterior *Rok-RNAi* (K) mitotic cells, at each developmental stage. Black dashed line represents region enlarged in Figure 5B-E. (L) Mitotic nuclear velocity for control and *Rok-RNAi* expressing mitotic cells, at each developmental stage. Calculated from onset of apical movement and cortical mitotic enrichment, up to most-apical position, i.e. metaphase. For 72hr, 96hr and 120hr wildtype cells, the mean onset of apical movement was -11.1, -10.0 and -12.1 min respectively and the duration was 9.2, 8.0 and 9.4 min respectively. For 72hr, 96hr and 120hr *Rok-RNAi* expressing cells, the mean onset of apical movement was -9.9, -12.7 and -13.6 min respectively and the duration was 8.8, 11.8 and 12.1 min respectively. (M) Nuclear density per μm^3 for anterior control and posterior *Rok-RNAi* expressing wing disc regions, in Sqh-GFP and His-RFP backgrounds, at different developmental stages. Density significantly changes for *Rok-RNAi* region between time-points although wildtype region has delayed development. (B, C) n = 3 (72hr), 5 (96hr) and 6 (120hr) wing discs. (D-H) n = 8 (72hr), 7 (96hr) and 5 (120hr) wing discs. (J-M) n = 19-33 nuclear trajectories acquired from 3 wing discs per developmental stage. Scale bars: A, G, 50 μm; I, 5 μm. Statistical significance: E-H , Kolmogorov-Smirnov comparison of cumulative distribution; D, independent t-test; C, L and M, one-way ANOVA. n.s. $p>0.05$, *$p<0.05$, **$p<0.01$, ***$p<0.001$, ****$p<0.0001$. Error bars: E,G, median ± interquartile range; C, D, L, M, mean ± SD; J, K, mean ± SEM.

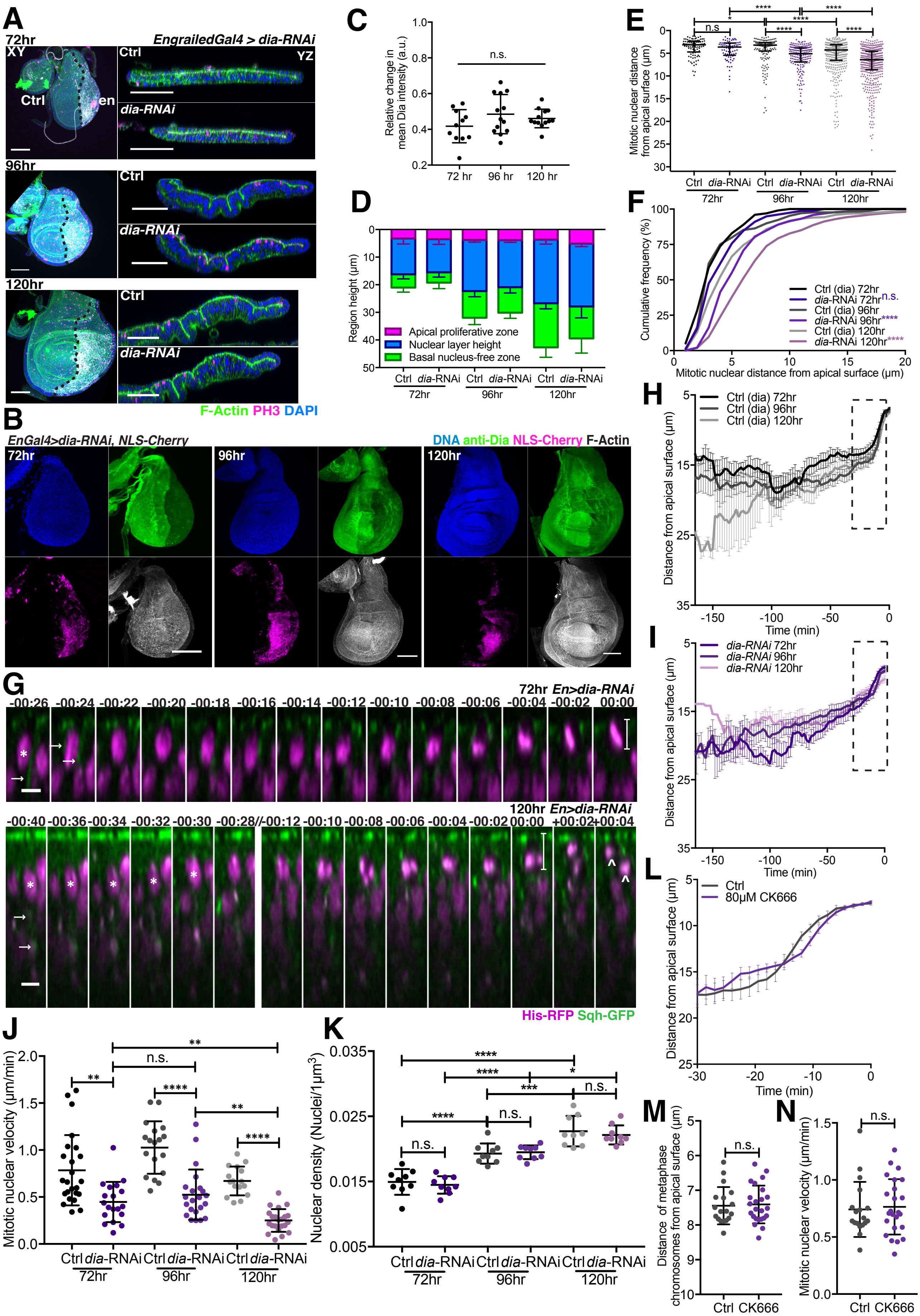

A
72hr
EngrailedGal4 > dia-RNAi
XY
Ctrl
YZ
Ctrl
en
dia-RNAi
96hr
Ctrl
dia-RNAi
120hr
Ctrl
dia-RNAi
F-Actin PH3 DAPI
B
EnGal4>dia-RNAi, NLS-Cherry
DNA anti-Dia NLS-Cherry F-Actin
72hr
96hr
120hr
C
Relative change in mean Dia intensity (a.u.)
n.s.
72 hr
96 hr
120 hr
D
Region height (µm)
Apical proliferative zone
Nuclear layer height
Basal nucleus-free zone
Ctrl dia-RNAi Ctrl dia-RNAi Ctrl dia-RNAi
72hr
96hr
120hr
E
Mitotic nuclear distance from apical surface (µm)
n.s
Ctrl dia-RNAi Ctrl dia-RNAi Ctrl dia-RNAi
72hr
96hr
120hr
F
Cumulative frequency (%)
Ctrl (dia) 72hr
dia-RNAi 72hr n.s.
Ctrl (dia) 96hr
dia-RNAi 96hr ****
Ctrl (dia) 120hr
dia-RNAi 120hr ****
Mitotic nuclear distance from apical surface (µm)
G
72hr En>dia-RNAi
-00:26 -00:24 -00:22 -00:20 -00:18 -00:16 -00:14 -00:12 -00:10 -00:08 -00:06 -00:04 -00:02 00:00
120hr En>dia-RNAi
-00:40 -00:36 -00:34 -00:32 -00:30 -00:28//-00:12 -00:10 -00:08 -00:06 -00:04 -00:02 00:00 +00:02 +00:04
His-RFP Sqh-GFP
H
Ctrl (dia) 72hr
Ctrl (dia) 96hr
Ctrl (dia) 120hr
Distance from apical surface (µm)
Time (min)
I
dia-RNAi 72hr
dia-RNAi 96hr
dia-RNAi 120hr
Distance from apical surface (µm)
Time (min)
J
Mitotic nuclear velocity (µm/min)
n.s.
Ctrl dia-RNAi Ctrl dia-RNAi Ctrl dia-RNAi
72hr
96hr
120hr
K
Nuclear density (Nuclei/1µm³)
n.s.
Ctrl dia-RNAi Ctrl dia-RNAi Ctrl dia-RNAi
72hr
96hr
120hr
L
Ctrl
80µM CK666
Distance from apical surface (µm)
Time (min)
M
Distance of metaphase chromosomes from apical surface (µm)
n.s.
Ctrl CK666
N
Mitotic nuclear velocity (µm/min)
n.s.
Ctrl CK666

Figure S6. Dependency on Dia for apical mitotic nuclear movement increases with development. Related to Figure 6.

(A) Representative images of wing disc morphology with *dia-RNAi* expression in *engrailed* domain (posterior, *en*) at different developmental stages. *dia-RNAi* expression marked by UAS-NLS-Cherry in the posterior region (grey), phalloidin (green), anti-PH3 (magenta) and DAPI (blue). Black dashed line shows anterior-posterior boundary. (B) Representative images of anti-Dia staining (green) in *EnGal4>dia-RNAi* wing discs with DAPI (blue), UAS-NLS-Cherry (magenta) and F-actin (grey). (C) Relative change in anti-Dia staining in *dia-RNAi* region compared to control region. (D) Epithelial morphology measurements for control and *dia-RNAi* wing discs at each developmental stage. (E, F) Mitotic nuclear distance from apical surface for control and *dia-RNAi* wing discs presented as dot plot (E) and cumulative frequency distribution (F). (G) Representative images of *dia-RNAi* mitotic nuclei moving apically within posterior pouch region at 72 and 120 hours AEL. Wing discs express Sqh-GFP, His-RFP and *EnGal4>UAS-dia-RNAi*. Arrows: cortical myosin enrichment regions. Asterisks: tracked nuclei. Arrow heads: apico-basal cell division. Line bar: distance of metaphase nucleus from apical surface at 00:00 mins, with values presented in Figure 6I. (H, I) Full time course for average nuclear tracks for anterior control (H) and posterior *dia-RNAi* (I) mitotic cells, at each developmental stage. Black dashed line represents region enlarged in Figure 6B-E. (J-K) Mitotic nuclear velocity, calculated from onset of apical movement and cortical mitotic enrichment, up to most-apical position, i.e. metaphase (J), and nuclear density per μm^3 (K) for anterior control and posterior *dia-RNAi* expressing wing disc regions in Sqh-GFP and His-RFP backgrounds, at different developmental stages. For calculating the mitotic nuclear velocity, for 72hr, 96hr and 120hr wildtype cells, the mean onset of apical movement was -10.2, -9.8 and -9.3 min respectively and the duration was 7.1, 5.8 and 7.5 min respectively. For 72hr, 96hr and 120hr *dia-RNAi* expressing cells, the mean onset of apical movement was –13.7, -9.9 and -9.9 min respectively and the duration was 9.6, 7.7 and 8.7 min respectively. (C) n = 11 (72hr), 13 (96hr), 13 (120hr) wing discs. (D-F) n = 4 (72hr), 7 (96hr) and 8 (120hr) wing discs. (H-K) n = 16-24 nuclear trajectories acquired from 3-4 wing discs per developmental stage. Scale bars: A, E, 50μm, G, 5μm. Statistical significance: C, J, K; one-way ANOVA; E, Kolmogorov-Smirnov comparison of cumulative distribution. n.s. $p>0.05$, *$p<0.05$, **$p<0.01$, ****$p<0.0001$. Error bars: C, J, K, mean ± SD; E, median ± interquartile range; H, I, mean ± SEM.

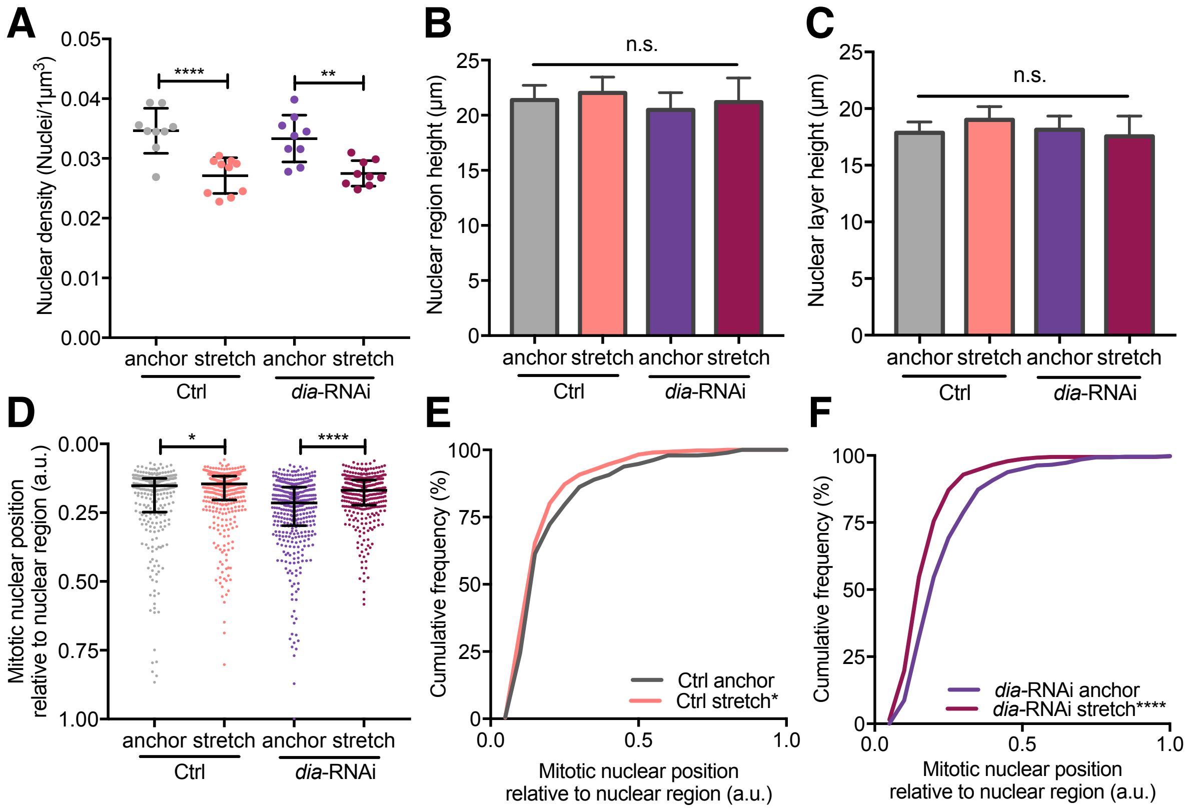

Figure S7. Defects in nuclear positioning upon Dia depletion can be rescued by mechanically reducing cell density. Related to Figure 7.

(A-C) Density of nuclei per 1μm^3 (A), nuclear region height (B), and nuclear layer height (C), in pouch region for control and *dia-RNAi* expressing discs in anchor and stretched conditions. (D-E) Mitotic nuclear position relative to the nuclear region for control (E) and *dia-RNAi* expressing (F) wing disc pouch regions in anchor and stretched conditions presented as dot plot (D) and cumulative frequency distribution (E, F). Data corresponds to Figure 7B-D. (A) n = 3 wing discs per condition. (B-F) n = 7 (anchored) and 9 (stretched) control wing discs, and n = 9 (anchored) and 8 (stretched) *dia-RNAi* wing discs. Statistical significance: A, unpaired t-test; B, C, one-way ANOVA; D-F, Kolmogorov-Smirnov comparison of cumulative. n.s. p>0.05, *p<0.05, **p<0.01, ****p<0.0001. Error bars: A-C, mean ± SD; D, median interquartile range.
